# Molecular Signaling Pathways of Quercetin in Alzheimer’s Disease: A Promising Arena

**DOI:** 10.1007/s10571-024-01526-w

**Published:** 2024-12-24

**Authors:** Mansour A. Alsaleem, Hayder M. Al-kuraishy, Ali I. Al-Gareeb, Ali K. Albuhadily, Mohammed Alrouji, Asmaa S. A. Yassen, Athanasios Alexiou, Marios Papadakis, Gaber El-Saber Batiha

**Affiliations:** 1 Unit of Scientific Research, Applied College, Qassim University, Qassim, Saudi Arabia; 2 Department of Clinical Pharmacology and Medicine, College of Medicine, Mustansiriyah University, Baghdad, Iraq; 3 Department of Clinical Pharmacology and Medicine, Jabir Ibn Hayyan Medical University, Kufa, Iraq; 4 Department of Clinical Laboratory Sciences, College of Applied Medical Sciences, Shaqra University, Shaqra, 11961 Saudi Arabia; 5 Department of Medicinal Chemistry, Faculty of Pharmacy, Galala University, New Galala City, Suez, 43713 Egypt; 6 Pharmaceutical Organic Chemistry Department, Faculty of Pharmacy, Suez Canal University, Ismailia, 41522 Egypt; 7 University Centre for Research & Development, Chandigarh University, Chandigarh-Ludhiana Highway, Mohali, Punjab India; 8 Department of Science and Engineering, Novel Global Community Educational Foundation, Hebersham, NSW 2770 Australia; 9 Department of Research and Development, Funogen, 11741 Athens, Greece; 10 University Hospital Witten-Herdecke, University of Witten-Herdecke, Heusnerstrasse 40, 42283 Wuppertal, Germany; 11 Department of Pharmacology and Therapeutics, Faculty of Veterinary Medicine, Damanhour University, Damanhour, 22511 AlBeheira Egypt

**Keywords:** Alzheimer’s disease, Quercetin, Neurodegenerative diseases, Neuroprotective effect, Oxidative stress, Molecular mechanism

## Abstract

Alzheimer’s disease (AD) is a neurodegenerative disease characterized by cognitive impairment and memory deficit. Even with extensive research and studies, presently, there is no effective treatment for the management of AD. Besides, most of drugs used in the treatment of AD did not avert the AD neuropathology, and the disease still in a progressive status. For example, acetyl cholinesterase inhibitors are associated with many adverse effects, such as insomnia and nightmares. As well, acetylcholinesterase inhibitors augment cholinergic neurotransmission leading to the development of adverse effects related to high acetylcholine level, such as salivation, rhinorrhea, vomiting, loss of appetite, and seizure. Furthermore, tacrine has poor bioavailability and causes hepatotoxicity. These commonly used drugs do not manage the original causes of AD. For those reasons, natural products were repurposed for the treatment of AD and neurodegenerative diseases. It has been shown that phytochemicals produce neuroprotective effects against the development and progression of neurodegenerative diseases by different mechanisms, including antioxidant and anti-inflammatory effects. Quercetin (QCN) has been reported to exert an effective neuroprotective effect against AD and other neurodegenerative diseases by lessening oxidative stress. In this review, electronic databases such as PubMed, Scopus, and Web of Science were searched for possible relevant studies and article linking the effect of QCN on AD. Findings from this review highlighted that many studies highlighted different mechanistic signaling pathways regarding the neuroprotective effect of QCN in AD. Nevertheless, the precise molecular mechanism of QCN in AD was not completely clarified. Consequently, this review aims to discuss the molecular mechanism of QCN in AD.

## Introduction

Alzheimer’s disease (AD) is a neurodegenerative disease, linked with the development of cognitive impairment, memory, and neuropsychiatric disorders (Scheltens et al. 2021). AD accounts for more than two-third of dementia in old age subjects (Li et al. 2021 Bhatia et al. 2023). Of note, there are two types of AD, sporadic AD is account for 90% of AD, and familial AD is account for 10% of AD. Sporadic AD is more correlated with old age > 65 year and late-onset AD; however, familial AD is related with the development of early-onset AD (Jellinger 2020). AD neuropathology is characterized by the progressive deposition of extracellular amyloid-beta protein (Aβ) and intracellular accumulation of hyperphosphorylated tau protein (Trejo-Lopez et al. 2022). Increasing the accumulation of Aβ is due to either overproduction of Aβ from mutant amyloid precursor protein (APP) or defect in the clearance of Aβ (Li et al. 2020). In normal physiological condition, most of APP processing chiefly in young is through the non-amyloidogenic pathway to produce the neuroprotective soluble APP alpha (sAPPα) (Dar and Glazner 2020; Pfundstein et al. 2022). The amyloidogenic pathway is primarily mediated by γ and β secretases; however, the non-amyloidogenic pathway is mediated by α secretase. Nevertheless, in aging and chronic inflammatory and oxidative stress disorders, APP processing is shifted toward the amyloidogenic pathway to produce the neurotoxic Aβ1–42 which triggers inflammation and neuronal apoptosis (Dar and Glazner 2020; Pfundstein et al. 2022). Furthermore, tau protein is normally present in the healthy brain and intricate in the regulation of axonal transport and microtubule stabilization (Muralidar et al. 2020). However, genetic mutation of tau protein gene or activation of inflammatory signaling pathway induces hyperphosphorylation of tau protein (Basheer et al. 2023). Buildup of hyperphosphorylated tau protein which form neurofibrillary tangle (NFT) is related with progressive neuronal injury and the development of AD (Basheer et al. 2023; Muralidar et al. 2020). Importantly, Aβ and NFT interacts together to induce inflammation and oxidative stress which promote neurodegeneration in AD (Zhang et al. 2021). Moreover, the major pathological hallmarks, NFT and Aβ plaques can result from dysfunctional insulin signaling. Insulin is an important growth factor that regulates cell growth, energy utilization, mitochondrial function, autophagy, oxidative stress, synaptic plasticity, and cognitive function. Insulin and its downstream signaling molecules are located majorly in the regions of cortex and hippocampus. The major molecules involved in impaired insulin signaling include IRS, PI3K, Akt, and GSK-3β (Akhtar and Sah 2020; Kumar et al. 2020). Activation or inactivation of these major molecules through increased or decreased phosphorylation plays a role in insulin signaling abnormalities or brain insulin resistance (IR). IR is considered as a major culprit in generating the hallmarks of AD arising from neuroinflammation and oxidative stress. Moreover, caspases, Nrf2, and NF-κB influence this pathway in an indirect way. Various studies also suggest a strong link between type 2 diabetes (T2D) and AD due to the impairment of BRAIN insulin signaling pathway (Akhtar and Sah 2020). Insulin might be involved in the pathogenesis of AD which coined as a type 3 diabetes (T3D). The nuclear factor erythroid 2-related factor 2 (Nrf2) triggers a cascade of events under the regulation of distinct mechanisms, including protein stability, phosphorylation, and nuclear cytoplasmic shuttling, finally leading to the protection against oxidative damage in AD. In addition, there is a strong correlation between insulin and Nrf2 signaling pathways both in the periphery and the brain but merely few of them have focused on elucidating their inter-connective role in AD (Rahman et al. 2023).

**Fig. 1:**
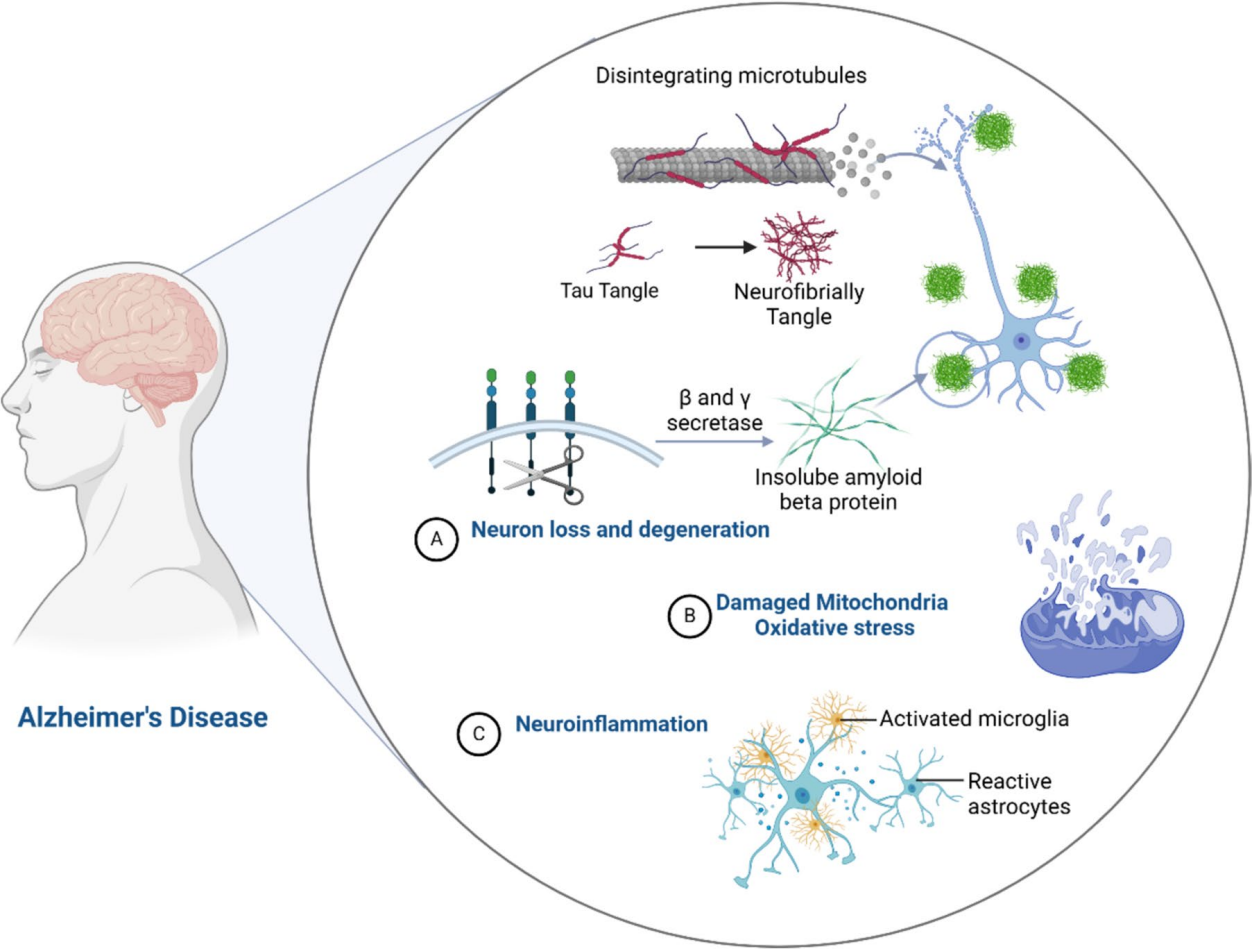
Alzheimer’s disease neuropathology

Therefore, AD neuropathology is multifarious and related to dissimilar cellular and sub-cellular disorders, such as autophagy dysfunction, mitochondrial dysfunction, oxidative stress, and neuroinflammation (Jurcău et al. 2022) (Fig. 1).

Despite of extensive researches and studies, currently, there is no single effective treatment of AD (Yiannopoulou and Papageorgiou 2020). In addition, most of the drugs used in the symptomatic treatments of AD did not avert the neuropathology, and the disease is still in a progressive status. Moreover, acetylcholinesterase inhibitors are associated with the development of severe adverse effects, such as diarrhea, nausea, abdominal pain, insomnia, and nightmares (Kim et al. 2010). As well, acetylcholinesterase inhibitors augment cholinergic neurotransmission leading to the development of adverse effects related to high acetylcholine level, such as salivation, rhinorrhea, vomiting, loss of appetite, and seizure (Kim et al. 2010). In addition, tacrine has poor bioavailability and cause hepatotoxicity. Likewise, memantine is less effective than acetylcholinesterase inhibitors in the management of AD (Kim et al. 2010). These commonly used drugs do not manage the root causes of AD. For that reasons, natural products were repurposed for the treatment of AD and neurodegenerative diseases (Sharifi-Rad et al. 2020). It has been shown that phytochemicals produce neuroprotective effects against the development and progression of neurodegenerative diseases by different mechanisms, including antioxidant and anti-inflammatory effects (Sharifi-Rad et al. 2020). For example, Quercetin (QCN) has been reported to exert a neuroprotective effective effect against AD and neurodegenerative diseases by alleviating oxidative stress (Bayazid and Lim 2022). Furthermore, many studies highlighted different mechanistic signaling pathways regarding the neuroprotective effect of QCN in neurodegenerative diseases (Grewal et al. 2021; Yu et al. 2020). Importantly, QCN has previously been discussed against neurotoxicity and neurodegenerative diseases, including AD, in numerous previous reports. However, the exact molecular mechanism of QCN in AD was not fully elucidated. Therefore, this review aims to discuss the molecular mechanism of QCN against the development and progression of AD.

### QCN Overview

**Fig. 2:**
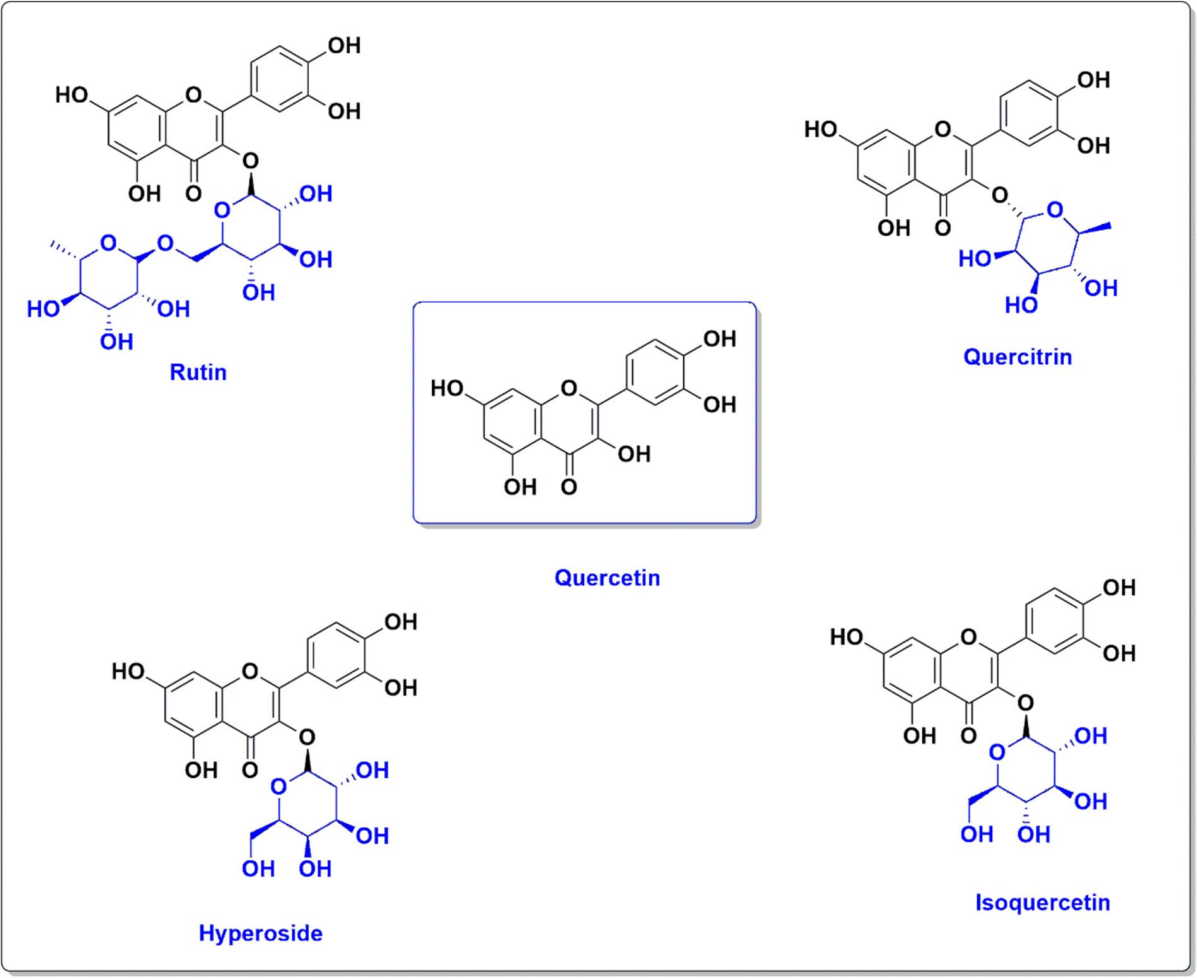
Chemical structure of quercetin and its derivatives

QCN is a polyphenol plant found in different vegetables, fruits, seeds and red onion (P. Singh et al. 2021). QCN is broadly distributed in nature; its name was derived from oak forest (*quercetum*) since 1857 (Yang et al. 2020) (Fig. 2).

**Fig. 3:**
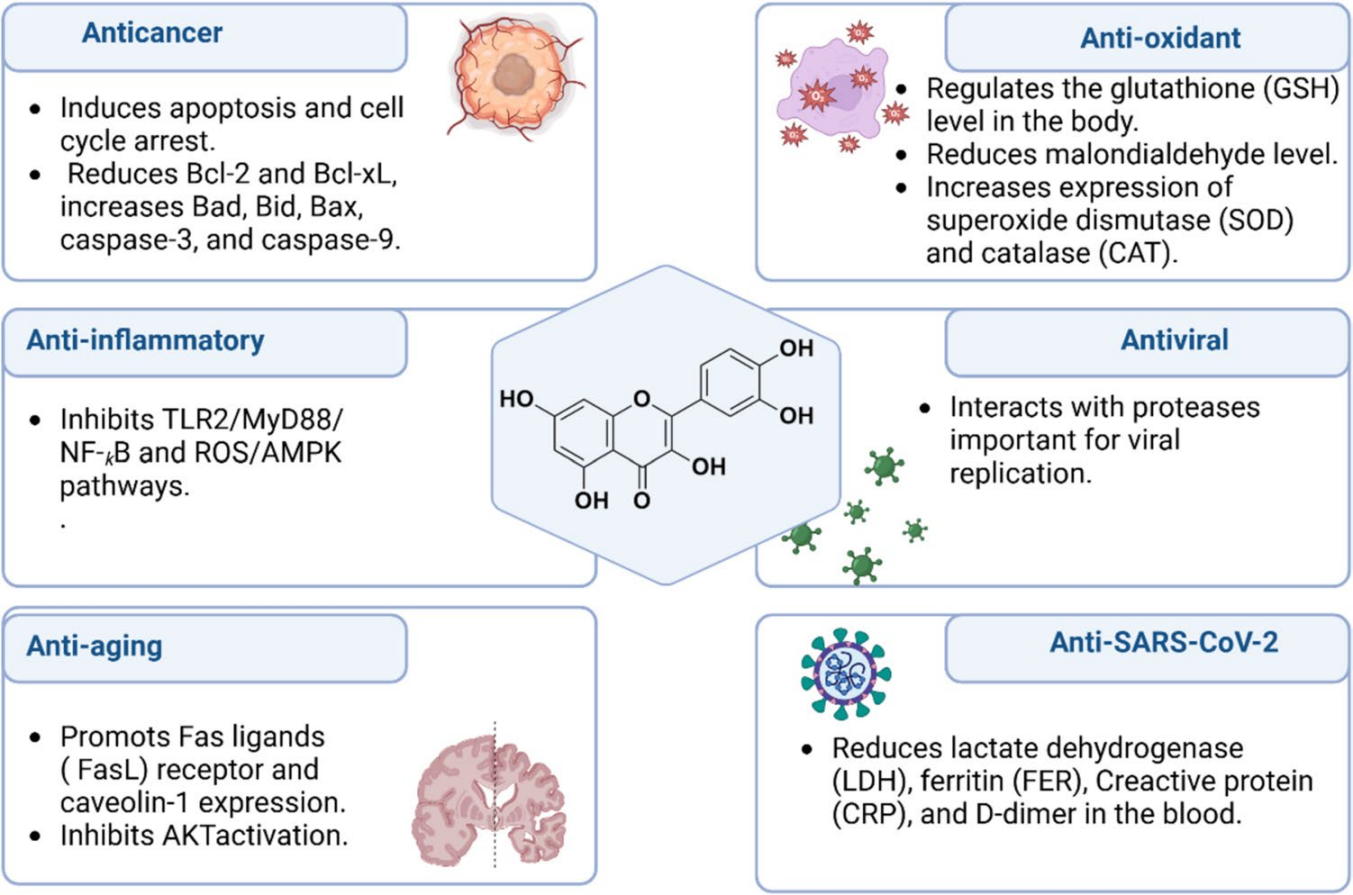
Pharmacological effects of quercetin

Of note, QCN has poor bioavailability < 1% after oral intake due to poor solubility, as well; intravenous administration of QCN is rapidly metabolized by liver in a dose-dependent manner (Batiha et al. 2020). Therefore, findings from in vitro studies do not correspond with the in vivo studies. However, intake of QCN with high-fat diet improves it absorption. QCN inhibits metabolism of many medications via inhibition of hepatic cytochrome (Batiha et al. 2020; Yang et al. 2020). However, QCN is safe in animals and humans, although its safety during pregnancy and breast feeding was not confirmed exactly (Batiha et al. 2020; Yang et al. 2020). In virtue of its anti-inflammatory, antioxidant, an antimicrobial, and antitumor activity, QCN is widely used as adjuvant treatment in treating inflammatory and oxidative disorder, infections, malignancy and metabolic, and cardiovascular diseases by modulating many signaling pathway (Wang et al. 2022a, b) (Fig. 3).

## Neuroprotective Effect of QCN

It has been shown that QCN has a neuroprotective role against the development of neurodegenerative diseases such as AD and Parkinson’s disease (PD) by inhibiting the neuroinflammation (Khan et al. 2018; Kalra et al. 2024). Supporting to this claim, QCN attenuates cognitive impairment and memory deficit in lipopolysaccharide (LPS)-induced neuroinflammation by suppressing the release of pro-inflammatory cytokines in mice (Khan et al. 2018). Moreover, QCN inhibits the activation of apoptotic pathway through modulation Bax/Bcl-2/capspase-3 signaling in the hippocampus and cerebral cortex in mice with LPS-induced neuroinflammation and neurotoxicity (Khan et al. 2018). In addition, QCN has beneficial effects against many neurological and neuropsychiatric diseases such as attention deficit hyperactivity disorders (Alvarez-Arellano et al. 2020; Dvořáková et al. 2006). As well, QCN is responsible for antidepressant like effects of *Allium cepa* outer scale extract possibly via prevention of brain oxidative stress and restoring serotonin levels by inhibiting the activity of monoamine oxidase A (MAO-A) (Singh et al. 2021; Kumar et al. 2020). It has been shown that hyperactivation of MAO results in the generation of ROS, which leads to a variety of neurological diseases, such as AD. However, MAO inhibitors are linked with side effects, such as hepatotoxicity and hypertensive crisis, necessitating the investigation of alternative MAO inhibitors from a natural source with a safe profile. QCN is regarded as a potential MAO inhibitor that could be effective in the management of AD and other neurological disorders (Mannan et al. 2022). In addition, a QCN derivative trimethoxy flavone is responsible for the memory improvement effect of *Ocimum basilicum L*. leave extract due to their anticholinergic, antioxidant, anti-inflammatory, and anti-apoptotic properties (Singh et al. 2021). These maybe developed as valuable alternatives for management of cognitive disorders. Ardianto et al. (Ardianto et al. 2023) found that intraperitoneal administration of QCN reduces stroke-induced cognitive and memory deficit in mice through modulation the expression and the activity of glutamatergic neurotransmission and melanocortin signaling in the hippocampus. A systematic review and meta-analysis highlighted that QCN attenuates experimental ischemic stroke and could be effective treatment focal cerebral ischemia (Guo et al. 2022). Moreover, QCN can reduce PD neuropathology by inhibiting oxidative stress and inflammation in the dopaminergic neurons of the substantia nigra pars compacta (SNpc) (Tamtaji et al. 2020). PD is regarded as a second common neurodegenerative disease due to the accumulation of alpha synuclein (αSyn) in the dopaminergic neurons of the SNpc and associated neuronal apoptosis (Al-kuraishy et al. 2024a, b, c; Al-kuraishy et al. 2023a, b). Wang et al., (Wang et al. 2021a, b, c) illustrated that QCN protects dopaminergic neurons of the SNpc from the neurotoxic effect of 6-OHDA in PD model by preventing the development of mitochondrial dysfunction. Besides, QCN decreases the expression of α-Syn protein by inhibiting oxidative stress and mitochondrial dysfunction in PC12 cells subjected to the 6-OHDA (Wang et al. 2021a, b, c). Similarly, QCN attenuates apoptosis of the dopaminergic neurons in the SNpc by suppressing ferroptosis in MPTP (1-methyl-4-phenyl-1, 2, 3, 6-tetrahydropyridine) PD mouse model (Lin et al. 2022). Ferroptosis is a programmed cell death caused the accumulation of iron and lipid peroxide. QCN inhibits ferroptosis through activation of Nrf2 proteins which has antioxidant and prevents oxidative stress and lipid peroxidation (Lin et al. 2022). As well, QCN attenuates vascular dementia-induced neuropsychiatric disorders by improving cerebral blood flow and inhibiting microglia both in vitro and in vivo (Tan et al. 2022). Furthermore, QCN has a neuroprotective effect against the development of amyotrophic lateral sclerosis (ALS) which is a progressive neurodegenerative of motor neurons (Al-kuraishy et al. 2024c; Jin et al. 2023). QCN reduces the pathogenesis of ALS by inhibiting inflammation and endoplasmic reticulum (ER) stress through activation the neuroprotective signaling proteins, including Sirtuin 1 (SIRT1), AMP-activated protein kinase (AMPK), and sestrin 2 (Jin et al. 2023). Lazo-Gomez et al., (Lazo-Gomez and Tapia 2017) highlighted that QCN alleviates neurodegeneration of motor neurons induced by chronic excitotoxicity through activation of SIRT1 signaling pathway.

Moreover, QCN has been reported to effective in the management of multiple sclerosis (MS) and other demyelinating diseases (Javanbakht et al. 2023). MS is a common autoimmune demyelinating disease of the white matter in both brain and the spinal cord. In addition, MS is associated with the development of neurodegeneration result in cognitive impairment (Al-kuraishy et al. 2024a, b, c; Alruwaili et al. 2023). QCN by inhibiting oxidative stress and inflammation prevents demyelination and enhances remyelination in animal models (Javanbakht et al. 2023). In addition, QCN modulates the immune response in experimental MS through inhibition the release of pro-inflammatory cytokines from activated Th17 cells (Ahmadi et al. 2023).

**Fig. 4:**
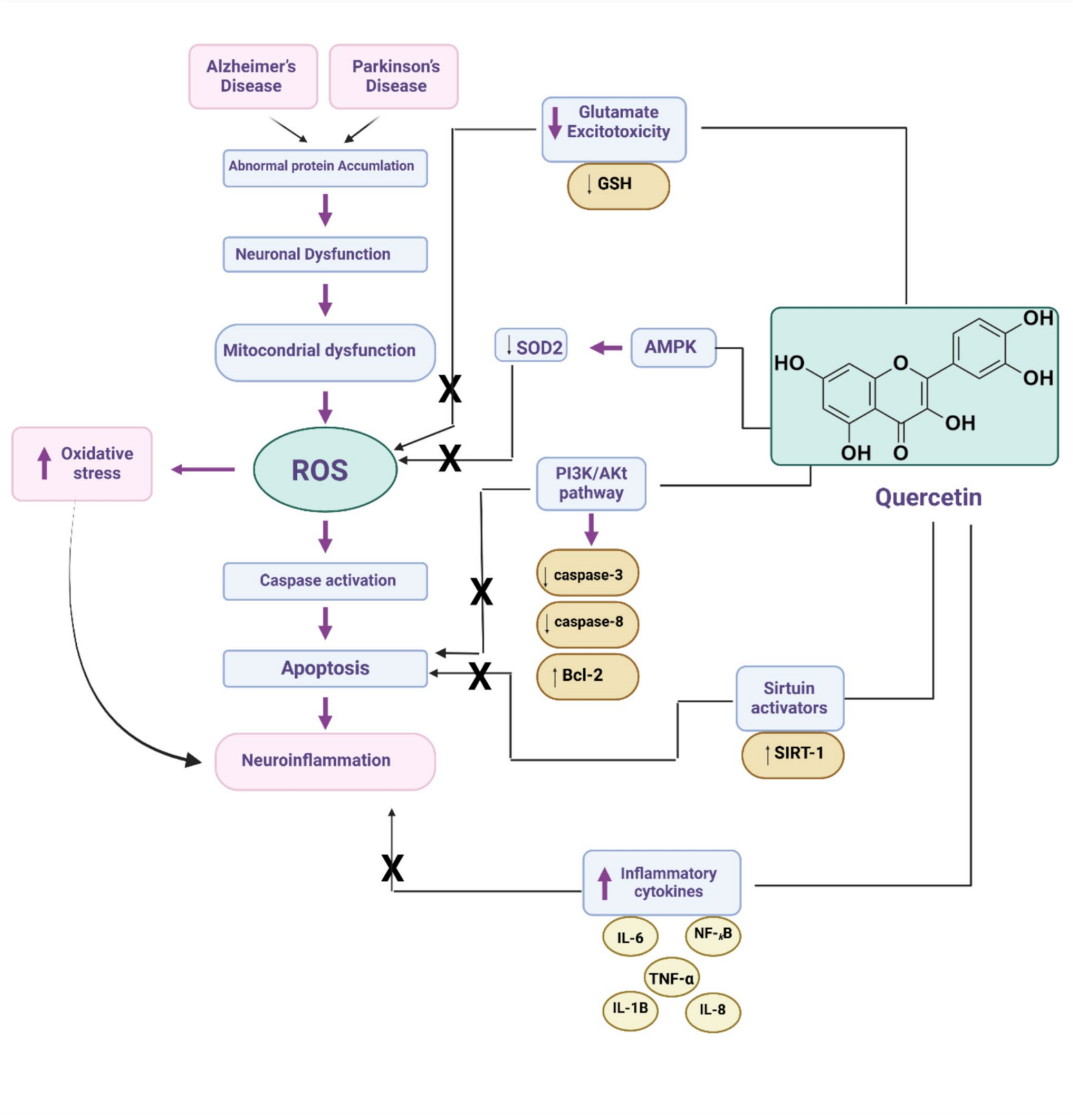
The neuroprotective effect of quercetin against neurodegenerative diseases

These findings indicated that QCN has a neuroprotective effect against different types of neurodegenerative diseases chiefly through suppression of oxidative stress, neuroinflammation, neurotoxicity, and other mechanisms (Fig. 4).

## Role of QCN in AD

Many studies highlighted that QCN ha a neuroprotective effect against the development and progression of AD (Table 1). QCN has been shown to inhibit the formation of amyloid plaque in the brain and associated inflammatory and oxidative stress disorders (Khan et al. 2019). QCN attenuates Aβ-induced neurotoxicity by inhibiting the generation of neurotoxic Aβ and linked oxidative stress and inflammation (Caruana et al. 2016). QCN mainly inhibits the formation of Aβ in the amyloidogenic pathway more efficiently than other herbal agents, such as datiscetin and kaempferol (Sato et al. 2013). It has been established that QCN inhibits Aβ formation through suppression of β-secretase (BACE-1) which responsible for the generation of the neurotoxic Aβ in the amyloidogenic pathway (Paris et al. 2011). Inhibition of BACE-1 by QCN is mediated by suppressing nuclear factor kappa B (NF-κB) which induce the expression of BACE-1 protein (Paris et al. 2011; Uddin et al. 2021). It has been shown that oral QCN nanoemulsion attenuates aluminum chloride-induced AD in animal model by regulating oxidative stress and inflammation (Alaqeel et al. 2022). Remarkably, QCN activates non-amyloidogenic pathway by activating the expression of α-secretase in AD rat model (Elfiky et al. 2021). Furthermore, QCN inhibits NFTs formation by reducing tau protein hyperphosphorylation in AD mouse model (Sabogal-Guáqueta et al. 2015). It has been reported that intraperitoneal administration of QCN (25 mg/kg) for 3 months inhibits tauopathy and amyloidosis in the hippocampus and attenuates cognitive deficit in aged transgenic mice (Sabogal-Guáqueta et al. 2015). A clinical trial illustrated that prolong intake of onion-rich QCN improves cognitive impairment and reduce risk of dementia (Nishimura et al. 2017). As well, 24-week intake of onion-rich QCN improves cognitive function in healthy elderly subjects (Nishihira et al. 2021).

**Table 1:** The neuroprotective effect of QCN in AD

Study type	Findings	References
Preclinical Preclinical Preclinical Preclinical Preclinical Preclinical Preclinical Preclinical Preclinical Preclinical Preclinical Clinical trial Clinical trial	QCN inhibits the formation of amyloid plaque in the brain and associated inflammatory and oxidative stress disorders QCN attenuates Aβ-induced neurotoxicity by inhibiting the generation of neurotoxic Aβ and linked oxidative stress and inflammation QCN inhibits BACE-1 which responsible for the generation of the neurotoxic Aβ QCN nanoemulsion attenuates aluminum chloride-induced AD in animal model by regulating oxidative stress and inflammation QCN activates non-amyloidogenic pathway by activating the expression of α-secretase in AD rat model QCN inhibits NFTs formation by reducing tau protein hyperphosphorylation in AD mouse model QCN (25 mg/kg) for 3 months inhibits tauopathy and amyloidosis in the hippocampus, and attenuate cognitive deficit in aged transgenic mice QCN reduce oxidative stress by inducing the expression of Nrf-2, PON2, and other antioxidant enzymes in rat model QCN improves mitochondrial biogenesis by reducing the generation of ROS in neuronal SH-SY5Y cells. QCN has a dose-dependent antioxidant effect by inhibiting COX-2 and iNOS in RA 264.7 cells QCN inhibits AChE in PC12 cells regardless of the interaction between Aβ and AChE QCN improves cognitive function by activating α7nAChR in experimental rodents. QCN has a neuroprotective effect against traumatic brain injury by remodeling of the gut-brain axis in mouse model A prolong intake of onion-rich QCN improves cognitive impairment and reduce risk of dementia A 24-week intake of onion-rich QCN improves cognitive function in healthy elderly subjects	Khan et al. (2019) Caruana et al. (2016) Paris et al. (2011) Alaqeel et al. (2022) Elfiky et al. (2021) Sabogal-Guáqueta et al. (2015) Sabogal-Guáqueta et al. (2015) Amanzadeh Jajin et al. (2021) Lee et al. (2024) Álvarez-Berbel et al. (2022), Liao et al. (2022) Singh and Garabadu (2021) Balasubramanian et al. (2023) Nishimura et al. (2017) Nishihira et al. (2021)

Moreover, QCN suppress the development of neuroinflammation which involved in the pathogenesis of AD (Adeoluwa et al. 2023; Al-Kuraishy et al. 2023). QCN inhibits LPS-induced depressive symptoms by inhibiting the activity of microglia in hippocampus of mouse model [49]. Therefore, QCN attenuates neuroinflammation-induced neurodegeneration in AD. Like other phytochemicals, QCN has a potent anti-inflammatory effect therefore can prevent the severity of inflammatory changes in AD (Adeoluwa et al. 2023). Findings from preclinical study demonstrated that QCN prevents the expression of inflammatory signaling pathways such as NF-κB and the release of pro-inflammatory cytokines such as TNF-α and IL-1β in LPS mouse model (Qureshi et al. 2011). Thus, QCN by inhibiting the release of pro-inflammatory cytokines and activating the release of anti-inflammatory cytokines can attenuate the development of neuroinflammation in AD.

Furthermore, QCN can reduce the development of oxidative stress which implicated in the pathogenesis of AD (Al-kuraishy et al. 2023a, b; Molaei et al. 2020). Oxidative stress contributes in the progression of AD neuropathology by activating the generation of Aβ. In turn, the neurotoxic Aβ triggers the expression of ROS and inhibits the expression of antioxidant proteins such as superoxide dismutase (SOD) (Al-kuraishy et al. 2023a, b; Butterfield 2014). QCN reduce oxidative stress by inducing the expression of Nrf-2, paraoxonase-2 (PON2) and other antioxidant enzymes in rat model (Amanzadeh Jajin et al. 2021). Thus, QCN can attenuate oxidative stress-induced neurodegeneration in AD (Molaei et al. 2020). Ho et al. (2022) found that QCN improves mitochondrial biogenesis by reducing the generation of ROS in neuronal SH-SY5Y cells. QCN has a dose-dependent antioxidant effect by inhibiting cyclooxygenase 2 (COX-2) and inducible nitric oxide synthase (iNOS) in RA 264.7 cells (Lee et al. 2024).

Of interest, cholinergic dysfunction and impairment of cholinergic neurotransmission is intricate in the development of cognitive impairment in AD (Sultzer et al. 2022). Therefore, augmentation of acetylcholine level by inhibiting of acetylcholinesterase (AChE) may be a therapeutic strategy in the managements of AD (Alber et al. 2020). Findings from an experimental study observed that the herbal medicine resveratrol and its derivative *P. florida* by inhibiting AChE could be a promising therapy for AD (Randhawa et al. 2021). Many studies highlighted that QCN has ability to binds and inhibits AChE in a concentration-dependent manner (Abdalla et al. 2013, 2014). QCN inhibits the activity of AChE in PC12 cells regardless of the interaction between Aβ and AChE (Álvarez-Berbel et al. 2022; Liao et al. 2022). Singh and Garabadu (2021) revealed that QCN improves cognitive function by activating α7nAChR in experimental rodents.

Interstingly, dysregulation of gut-brain axis signaling is involved in the pathogenesis of AD. The gut microbiota is a complex ecosystem that comprises of more than 100 trillion symbiotic microbial cells. The microbiota, the gut, and the brain form an association, the microbiota-gut-brain axis, and synchronize the gut with the CNS and modify the behavior and brain immune homeostasis (Doifode et al. 2021). The bidirectional communication between gut and brain occurs via the immune system, the vagus nerve, the enteric nervous system, and microbial metabolites, including short-chain fatty acids (SCFAs), proteins, and tryptophan metabolites. Recent studies have implicated the gut microbiota in many neurodegenerative diseases, including AD. Understanding the role of the microbiota may provide new targets for treatment to delay the onset, progression, or reverse AD and may help in reducing the prevalence of AD (Doifode et al. 2021). Alteration in the gut microbiota composition is determined by increase in the permeability of the gut barrier and immune cell activation, leading to impairment in the BBB function that promotes neuroinflammation, neuronal loss, neural injury, and eventually AD (Megur et al. 2021). Many studies have shown that the gut microbiota plays a crucial role in brain function and changes in the behavior of individuals and the formation of bacterial amyloids. LPS and bacterial amyloids synthesized by the gut microbiota can trigger the immune cells residing in the brain and can activate the immune response leading to neuroinflammation. Growing experimental and clinical data indicate the prominent role of gut dysbiosis and microbiota–host interactions in AD (Doifode et al. 2021; Megur et al. 2021). Therefore, modulation of the gut microbiota with antibiotics or probiotic supplementation may create new preventive and therapeutic options in AD. It has been illustrated that QCN has a neuroprotective effect against traumatic brain injury by remodeling of the gut-brain axis in mouse model (Balasubramanian et al. 2023). Furthermore, polyphenols in QCN are also advanced glycation end product (AGE) inhibitors and therefore exert neuroprotective effects in AD (Reddy et al. 2020). Therefore, gut bacterial metabolism of lignans and other dietary polyphenolic compounds results in the formation of neuroprotective polyphenols, some of which have enhanced BBB permeability. It is hypothesized that gut bacterial metabolism-derived polyphenols, when combined with the nanoparticle-based BBB-targeted drug delivery, may prove to be effective therapeutics for various neurological disorders, including AD (Reddy et al. 2020).

Of note, prolong use of anti-AD medications is associated with the development of adverse effects that limit their use. In addition, anti-AD medications become ineffective with progression of disease (Kim et al. 2010). However, QCN accordingly to safety data from preclinical and clinical studies seems to be safer than anti-AD medications when used in old age individuals. As well, these findings highlighted that QCN can alleviates the fundamental pathways that implicated in the pathogenesis of AD. Herein, QCN seems to be superior to anti-AD medications in the managment of AD. Further, clinical trials and prospective studies are warranted to confirm this claim.

**Fig. 5:**
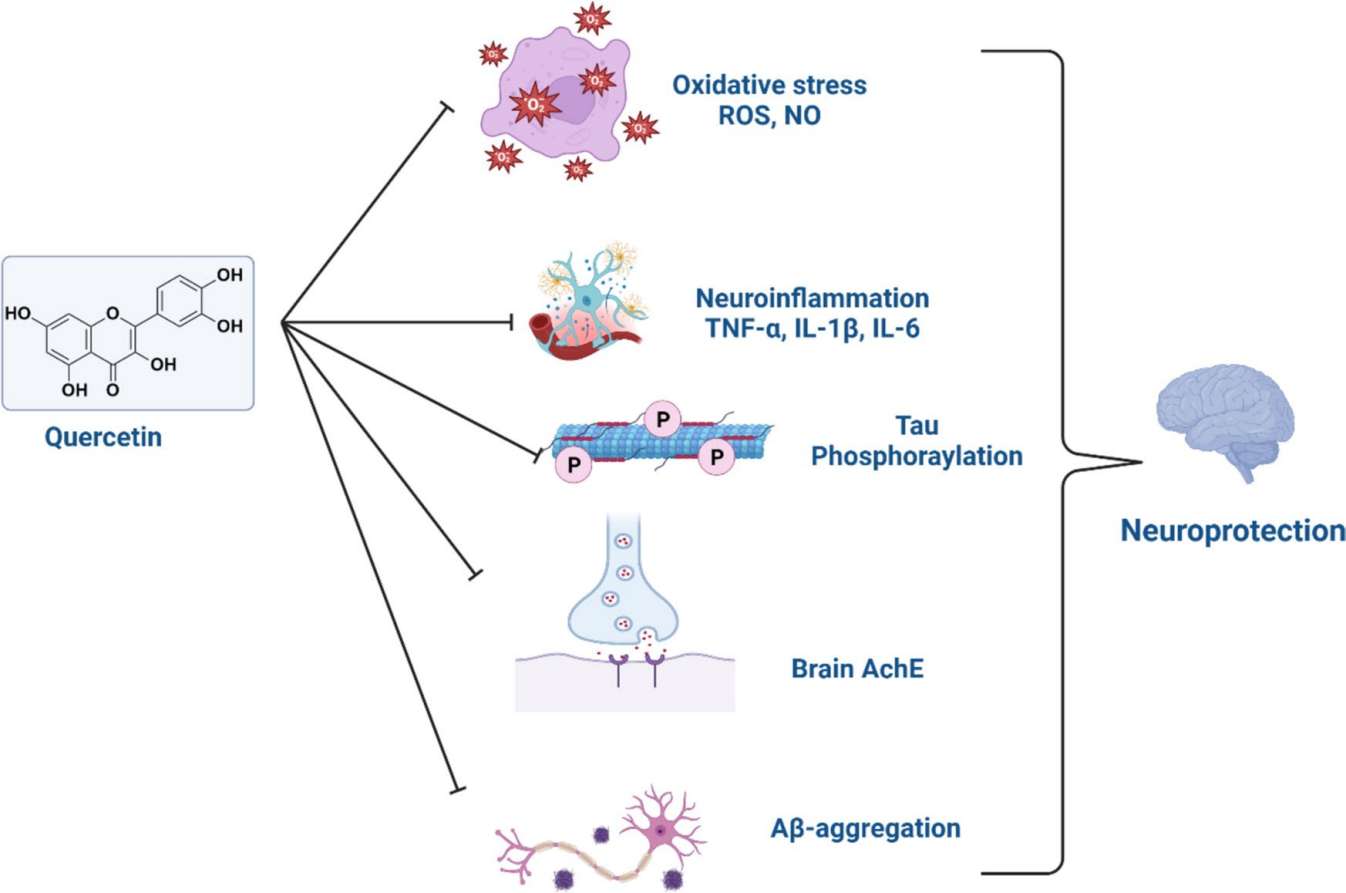
Role of quercetin against the development and progression of AD neuropathology

Taken together, QCN enhances cognitive impairment in AD through inhibition of Aβ and associated neuroinflammation and oxidative stress and suppression of brain AChE (Fig. 5).

## Mechanistic Role of QCN in AD Neuropathology

### Inositol Triphosphate 3-Kinase (PI3K)

Inositol triphosphate 3-kinase (IP3K) and protein kinase B (AKT) is a neuroprotective signaling pathway against the development and progression of neurodegenerative diseases, including AD (Kumar and Bansal 2022). It has been observed that downregulation of PI3K/AKT signaling pathway is involved in the pathogenesis of AD (Kumar and Bansal 2022). PI3K/AKT and mitogen activated protein kinase (MAPK) are essential molecules regulate brain insulin signaling (Zheng and Wang 2021). The PI3K/AKT signaling pathway regulates the activity of mechanistic target of rapamycin (mTOR), glycogen synthase kinase-3 beta (GSK3β), and cAMP-responsive element binding protein (CREB) that control neuronal survival, angiogenesis, mitochondrial function, metabolism, and autophagy (Zarneshan et al. 2022). It has been demonstrated that insulin and PI3K/AKT signaling pathway are reduced in the hippocampus of AD patients (Talbot et al. 2012) signifying that the development of brain IR is an initial incident in the development of AD. Aβ inhibits the expression and the activity PI3K/AKT signaling pathway results in neuronal apoptosis and progressive neurodegeneration in AD model (Zheng and Wang 2021; Kumar et al. 2020). In addition, Aβ indirectly inhibits PI3K/AKT signaling pathway via the activation of GSK3β and mTOR in mice (Choi et al. 2013). Likewise, augmented hyperphosphorylated tau protein inhibits the activity of PI3K/AKT by inducing the expression of GSK3β in AD rat model (Caccamo et al. 2011). A postmortem study found that the expression of PI3K/AKT was reduced in the frontal cortex in both T2D and AD patients (Liu et al. 2011) suggesting that T2D inhibits brain expression of PI3K/AKT and increase risk of AD. Correspondingly, findings from postmortem study established that the expression of the PI3K/AKT is reduced due to Aβ-induced activation in the brains of AD patients (Tramutola et al. 2015). Furthermore, PI3K/AKT signaling pathway regulates the expression of mitochondrial membrane permeability proteins Bax and Bcl-2 which intricate in the induction of apoptosis (Yang et al. 2022). Thus, deregulation of PI3K/AKT signaling pathway promotes AD neuropathology and activation of this pathway can prevent the development of brain IR and the progression of AD neuropathology. It has been shown that the neuroprotective effect of QCN against focal cerebral ischemia is mediated by activating PI3K/AKT signaling pathway and tropomyosin receptor kinase B (TrkB) receptor in rat model (Yao et al. 2012). Supporting to this finding, use of PI3K/AKT and TrkB receptor antagonists prevents the neuroprotective effect of QCN (Yao et al. 2012). Likewise, QCN attenuates the severity global cerebral ischemia in rat by stimulating PI3K/AKT signaling pathway and enhancement of anti-apoptotic pathway (LEI et al. 2015). QCN protects hippocampal neurons from apoptosis by activating PI3K/AKT signaling pathway in primary cortical neurons (Jiang et al. 2016). Moreover, QCN prevents the neurotoxicity induced by Aβ in HT22 cells through activation of PI3K/AKT signaling pathway (Jiang et al. 2016). Modulation of molecular dynamic of MAPK and PI3K/AKT signaling pathways attenuate AD neuropathology in T2D (Zu et al. 2021). Furthermore, QCN preserves blood brain barrier (BBB) integrity by activating PI3K/AKT signaling pathways in mice (Sun et al. 2024). These findings proposed that the neuroprotective effect of QCN against AD is mediated by activating PI3K/AKT signaling pathway.

### Glycogen Synthase Kinase-3 Beta (GSK3β)

GSK3β is a conserved threonine/serine kinase protein regulates different cellular metabolic pathways in response to the biological stimuli by activating the expression and the functional activity of glycogen synthase (Pecoraro et al. 2021). GSK3β promotes the synaptic plasticity, cognitive function, and neurodevelopment (Fan et al. 2020). GSK3β is distributed in all brain regions (Gizak et al. 2020). GSK3β modulates oxidative stress and DNA repair through regulation of Nrf2 expression (Fan et al. 2021). Many signaling involved in the regulation of GSK3β such as Wnt/β-catenin and PI3K are disturbed in neurodegenerative disease (Prossomariti et al. 2020). Nevertheless, exaggeration of GSK3β signaling pathway is intricate in the pathogenesis of AD and other neurodegenerative disease (Prossomariti et al. 2020).

Furthermore, the expression of GSK3β signaling pathway is augmented and involved in the pathogenesis of AD (Gadhave et al. 2021). As well, Aβ triggers the expression of GSK3β gene leading to synaptic failure and the development of dementia (Gupta et al. 2022). The activated GSK3β provokes the accumulation of Aβ and phosphorylated tau protein by activating the expression of BACE-1 (Song et al. 2022). Therefore, inhibition of GSK3β reduces the expression of *BACE-1* gene and reduces amyloid burden and associated inflammation (Fronza et al. 2023). Interestingly, GSK3β is negatively suppressed by insulin and insulin-like growth factor 1 (IGF-1), although the development of brain IR reduces the inhibitory effect of IGF-1 on the expression of GSK3β (Riis et al. 2020). A cohort study detected that stimulated GSK3β was augmented in the frontal cortex of AD patients (Leroy et al. 2007). Therefore, activation of GSK3β due to the development of brain IR or systemic inflammation overstates AD neuropathology. Activation of neuronal GSK3β by chronic systemic exposure to the LPS is developing in transgenic mice (M. Jiang et al. 2021). As well, several studies found that the activity of GSK3β was amplified in the peripheral blood of AD patients (Forlenza et al. 2011; Pláteník et al. 2014). Platelet GSK3β activity is increased in patients with cognitive impairment and AD (Forlenza et al. 2011). The activity of platelet GSK3β is linked with depressive symptoms and disease severity in AD patients (Pláteník et al. 2014).

It has been observed QCN reduces tau protein-induced oxidative stress by inhibiting GSK3β signaling in the hippocampal neurons (Jiang et al. 2016). QCN attenuates GSK3β signaling by activating the neuroprotective Wnt/β-catenin signaling in hippocampal neurons (Predes et al. 2022). In AD model, QCN produces a neuroprotective effect by inhibiting the expression of GSK3β signaling in the hippocampus (Zaplatic et al. 2019). Consistently, Chen et al., (Chen et al. 2016) illustrated that QCN improves cognitive function in AD mouse model by inhibiting ER stress via inhibition of GSK3β and MAPK signaling pathways. Thus, the neuroprotective effect of QCN against AD progression is mediated by inhibition of GSK3β signaling pathway.

### Protein Phosphatase 2A

Protein phosphatase 2A (PP2A) is a serine/threonine enzyme that induces dephosphorylation of tau protein and downregulation of PP2A is linked to tau protein hyperphosphorylation and the development of AD (Wei et al. 2020). Impairment of PP2A is associated with the development of sporadic AD due to hyperphosphorylation of tau protein and improvement in the phosphorylation of APP (Zhou et al. 2020). Moreover, mutation of Apolipoprotein E (ApoE4) inhibits the expression and the activity of PP2A in human brains of AD patients (Pires and Rego 2023). Noteworthy, PP2A activates neuronal autophagy and enhances the clearance of misfolded proteins. Suppression of PP2A leads to the inhibition of autophagy activity and exaggeration of AD neuropathology (Xu et al. 2021). It has been exemplified that sodium selenite increases the activity of PP2A in neuroblastoma cell line and in aged mice (Ahmed et al. 2020). Sodium selenite mitigates the cognitive function in transgenic mice by reducing tau protein level in the amygdala and hippocampus in transgenic mice by upregulating of PP2A activity (Zheng et al. 2016). Therefore, activation of the neuroprotective PP2A may reduce AD neuropathology.

It has been stated that QCN activates PP2A signaling and reduces the severity of brain injury. Findings from preclinical study demonstrated that QCN reduces neuronal damage in ischemic model and glutamate-induced neurotoxicity in HT22 cells by activating PP2A signaling pathway (PARK et al. 2019). Ansari et al., (Ansari et al. 2009) found that QCN prevents Aβ-induced neurotoxicity by activating the expression of PP2A in primary cortical neurons. Furthermore, QCN improves memory function in AD mouse model by activating the integrated stress response, such as PP2A (Nakagawa and Ohta 2019). These verdicts indicated that QCN by activating PP2A signaling pathway can improve memory and cognitive impairment in AD.

### Phosphatase and Tensin Homologue

Phosphatase and tensin homologue (PTEN) is phosphatase protein found in both nucleus and cytoplasm, intricate in the regulation of cell proliferation, growth, adhesion, apoptosis, and migration (Wang et al. 2021a, b, c). PTEN preserves the stability of DNA and chromosome genome through modulation transcription and translation of proteins by regulating PI3K/AKT which is inhibited by PTEN (Wang et al. 2022a, b). Therefore, PTEN is regarded as a tumor suppressor protein by inhibiting the excessive cellular growth by controlling the axonal growth in the CNS (Wang et al. 2021a, b, c; Wang et al. 2022a, b). It has been shown that inhibition of the expression of PTEN promotes neurite outgrowth in ischemic stroke model (Shen et al. 2020). Deletion of PTEN gene endorses synaptic plasticity and hippocampal excitatory synapses (Fraser et al. 2008). PTEN expression is altered in the postmortem brains of AD patients compared to controls (Sonoda et al. 2010). PTEN is mislocalized in the NFTs of AD patients leading to the inhibition of PI3K and PP2A. PTEN is implicated in the development of excitotoxicity and mitochondrial apoptosis in transgenic mice (Grande et al. 2014). It has been reported that APP promotes the expression of PTEN result in the inhibition of PP2A and the accumulation of tau protein in AD model (Goiran et al. 2018). In addition, hyperphosphorylated tau protein activates the expression of PTEN which induce neuronal loss and synaptic injury through activation of microglia in AD mouse model (Benetatos et al. 2020). Hence, mutant and overactivated PTEN is intricate in the pathogenesis of AD by inducing synaptotoxicity and tauopathy (Benetatos et al. 2020). Consequently, inhibition of PTEN may reduce AD neuropathology through PP2A-mediated synaptic plasticity and inhibition of tauopathy. It has been observed that PTEN inhibitor bisperoxovanadium (bpV) has a neuroprotective effect in many neurological disorders. Therefore, bisperoxovanadium may be effective against AD neuropathology (Mao et al. 2015). Additionally, plant sarcopoterium constrains the expression of PTEN and reduce the development and progression of AD (Ben-Shachar et al. 2019; He et al. 2021). These outcomes tinted that overexpression of PTEN is linked with AD neuropathology and thus inhibition of PTEN can attenuate the pathogenesis of AD.

Furthermore, QCN inhibits the expression of PTEN signaling pathway in male mice with prostatic cancer (Hao et al. 2024). Russo et al., illustrated QCN is regarded as a pleiotropic kinase inhibitor against cancer development (Russo et al. 2014). Moreover, QCN antagonizes mitochondrial dysfunction by inhibiting PTEN signaling pathway in hepatocytes (Miao et al. 2021). Therefore, the inhibition of PTEN expression by QCN promotes the activation of PP2A signaling pathway in AD with subsequent attenuation of the pathogenesis of AD neuropathology.

### Brain-Derived Neurotrophic Factor

Brain-derived neurotrophic factor (BDNF) is a neurotrophic protein acts by activating TrkB and p75 neurotrophin (p75NTR) receptors which are widely distributed the CNS (AlRuwaili et al. 2024). BDNF is released from neurons and peripheral tissue, although it is less cross the BBB (Ali, Al-kuraishy, et al. 2024a, b, c). BDNF has neuroprotective effect by enhancing synaptic plasticity and long-term potentiation (LTP) (Ali, et al. 2024a, b). Moreover, BDNF promotes hippocampal neurogenesis and attenuates the development of cognitive impairment (Turkistani et al. 2024). It has been shown that progression of AD neuropathology such as amyloid, NFTs, and associated neurodegeneration reduced the synthesis and the release of BDNF from neurons (Lim et al. 2022). Findings from preclinical study observed that the expression of BDNF mRNA is reduced in the hippocampus neurons of AD model (Mori et al. 2021). Consistently, the expression of BDNF mRNA is highly decreased in the parietal and entorhinal cortexes of the brains in AD patients (Girotra et al. 2022). In addition, low CSF BDNF is correlated with low expression of BDNF in the brains of AD patients (Girotra et al. 2022). Moreover, BDNF serum level is reduced in patients with dementia compared to healthy controls (Mori et al. 2021). These findings indicated that BDNF signaling mainly via TrkB is reduced and involved in the development of cognitive dysfunction in AD. Thus, activation of BDNF signaling could be a therapeutic strategy in the management of AD.

Notably, QCN attenuates cerebral ischemia mediated by activating TrkB receptor in rat model (Yao et al. 2012). Supporting to this finding, use of TrkB receptor antagonists prevent the neuroprotective effect of QCN (Yao et al. 2012). Furthermore, QCN has cardioprotective and antidepressant effects by activating of BDNF signaling in female mice (Wang et al. 2021a, b, c). In rat PD model, administration of QCN improves cognitive function and memory deficit by increasing the expression of hippocampal BDNF mRNA (Naghizadeh et al. 2021). In addition, QCN has a cognitive enhancer effect by enhancement of hippocampal antioxidant in PD rat model (Naghizadeh et al. 2021). Moreover, QCN prevents neuroinflammation and neuronal apoptosis by activating BDNF signaling in AD model (Alexander et al. 2023). Consistently, TrkB receptor agonist 7,8-dihydroxyflavone improves AD neuropathy by reducing the aggregation of Aβ in SH-SY5Y cells by increasing of BDNF-TrkB signaling pathway (Chiu et al. 2023). Thus, the neuroprotective effect of QCN against AD neuropathology may be through activation of BDNF-TrkB signaling pathway.

### Estrogenic Brain Signaling

Female sex hormone estrogen through the activation of estrogenic receptors which diffusely expressed in the brain contributes in the neuronal differentiation and improvement of behavior and synaptic plasticity (Maioli et al. 2021). Estrogenic receptor alpha (ERα) and estrogenic receptor beta (ERβ) have neuroprotective effects against neurodegenerative diseases, including AD and PD (Maioli et al. 2021). Estrogen exerts a neuroprotective effect from an early embryonic life to the aging through modulation of APP processing and the expression of BDNF signaling pathway (Wang et al. 2024). Mounting evidence suggests that estrogen replacement therapy improves AD outcomes in women with AD disease (Wang et al. 2024). A recent meta-analysis supports the neuroprotective role of estrogen against the development and the progression of neurodegenerative diseases, including AD and PD (Song et al. 2020). Thus, activation of brain estrogenic signaling may attenuate AD neuropathy.

Importantly, QCN is regarded as a phytoestrogen with identical chemical structure to that of estrogen (Liu et al. 2022). Therefore, QCN by activating brain ERα can attenuate Aβ-induced neurotoxicity. An in vitro study demonstrated that QCN reduced Aβ-induced neurotoxicity in PC12 cells by activating ERα/ERK1/2 signaling which inhibit the apoptotic signaling (Liu et al. 2022). In addition, QCN improve cognitive impairment in ovariectomized mice by stimulating the neuroprotective brain Erα (Aggarwal et al. 2020). Therefore, QCN through activation of estrogenic brain signaling could be a promising treatment for AD in postmenopausal women.

**Fig. 6:**
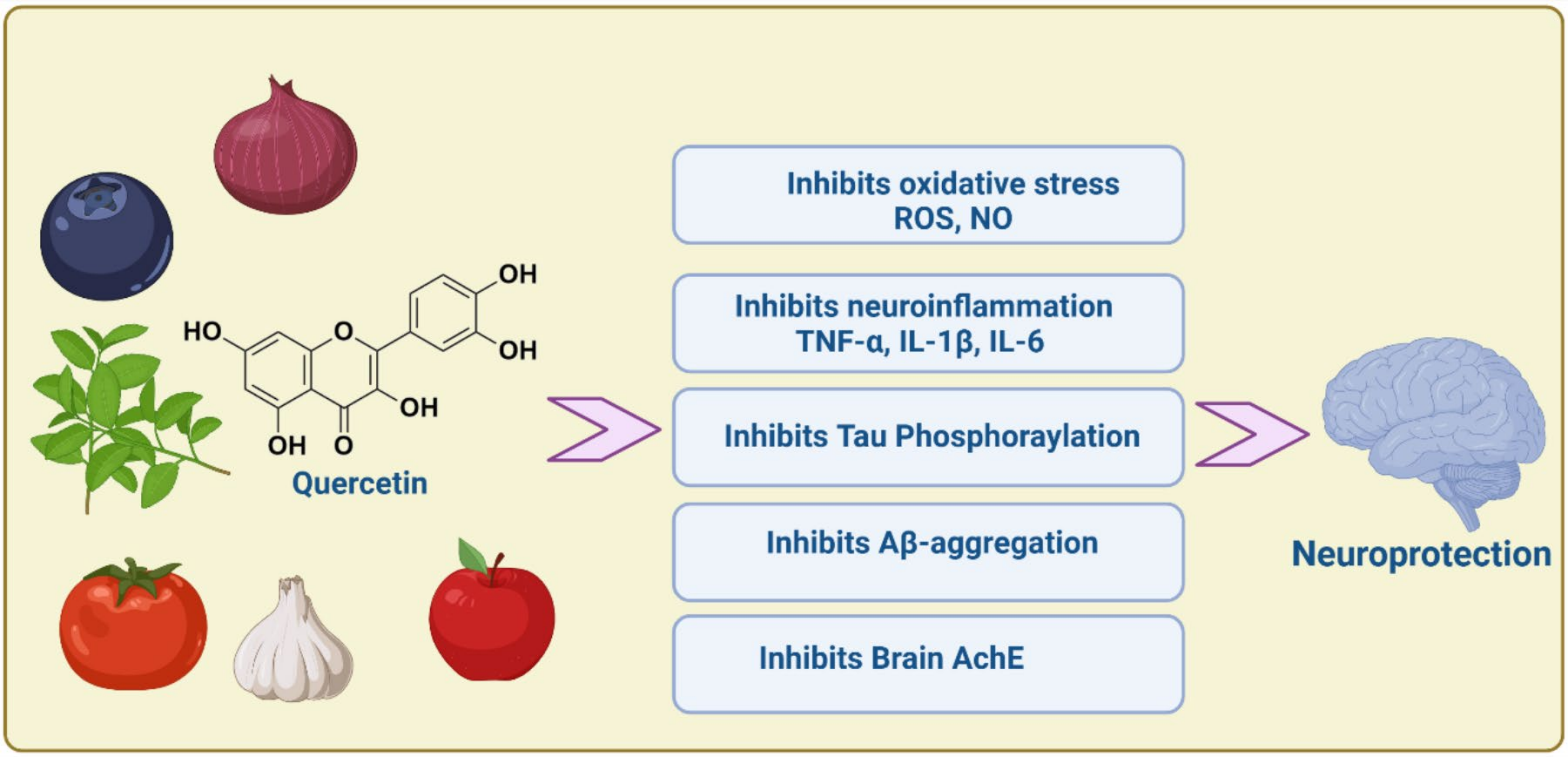
Mechanistic role of QCN against AD neuropathology

Taken together, QCN through modulation of different signaling pathways could be a promising therapeutic herbal remedy in the management of AD (Fig. 6, Table 2). However, QCN has poor bioavailability and crossing of the BBB. Therefore, different approaches were introduced to enhance QCN and crossing of the BBB (Alaqeel et al. 2022; Jaisamut et al. 2021). QCN nanoemulsion has higher bioavailability and can cross of the BBB (Mahadev et al. 2022). Thus, QCN nanoemulsion might be a novel therapeutic strategy in treating AD. Furthermore, the major obstacle in this field is the translation of preclinical knowledge into evidence-based clinical progress as preclinical findings are not correspondingly correlated with the clinical findings. Furthermore, the evaluation based on animal experiments data gave an objective judgment of the strength of evidence and the reliability of conclusions, which may guide the design of future preclinical tests and give supporting evidence for QCN as a promising drug in clinical treatment. Human trials of good quality are often missing or, when available, are frequently not suitable to really prove a therapeutically value (Fürst and Zündorf 2014). Thus, preclinical findings and limited clinical findings are the main limitations of this review. Importantly, the ability of QCN to reach the brain via BBB was not discussed in the different stages of AD that limits the therapeutic efficacy of QCN in pre-AD and symptomatic AD.

**Table 2:** The mechanistic role of QCN in AD

Study type	Findings	References
Preclinical Preclinical Preclinical Preclinical Preclinical Preclinical Preclinical Preclinical Preclinical Preclinical	QCN activates PI3K/AKT signaling pathway and TrkB receptor in rat model QCN preserves BBB integrity by activating PI3K/AKT signaling pathways in mice QCN reduces tau protein-induced oxidative stress by inhibiting GSK3β signaling in the hippocampal neurons QCN attenuates GSK3β signaling by activating the neuroprotective Wnt/β-catenin signaling in hippocampal neurons QCN reduces neuronal damage and glutamate-induced neurotoxicity in HT22 cells by activating PP2A signaling pathway QCN improves memory function in AD mouse model by activating the PP2A QCN inhibits the expression of PTEN signaling pathway QCN prevents neuroinflammation and neuronal apoptosis by activating BDNF signaling in AD model QCN by activating brain ERα can attenuate Aβ-induced neurotoxicity. QCN reduced Aβ-induced neurotoxicity in PC12 cells by activating ERα/ERK1/2 signaling which inhibit the apoptotic signaling QCN improve cognitive impairment in ovariectomized mice by stimulating the neuroprotective brain Erα receptor	Yao et al. (2012) Sun et al. (2024) Jiang et al. (2016) Predes et al. (2022) PARK et al. (2019) Nakagawa and Ohta (2019) Hao et al. (2024) Alexander et al. (2023) Liu et al. (2022) Aggarwal et al. (2020)

## Conclusion

Despite of extensive research and studies, currently, there is no single effective treatment for AD. In addition, most of drugs used in the symptomatic treatments of AD did not avert the neuropathology. For these reasons, natural products such as QCN were repurposed for the treatment of AD. QCN yields neuroprotective effects against the development and progression of neurodegenerative diseases by different mechanisms, including antioxidant and anti-inflammatory effects.

It has been shown that QCN has a neuroprotective role against AD by inhibiting the development of neuroinflammation. QCN has been shown to inhibit the formation of amyloid plaque in the brain and associated inflammatory and oxidative stress disorders. QCN attenuates Aβ-induced neurotoxicity by inhibiting the generation of neurotoxic Aβ and linked oxidative stress and inflammation. QCN mainly inhibits the formation of Aβ in the amyloidogenic pathway through suppression of BACE-1. Inhibition of BACE-1 by QCN is mediated by suppressing of NF-κB which induces the expression of BACE-1 protein. The neuroprotective effect of QCN against AD is mediated by activating PI3K/AKT, PP2A, BDNF, and estrogenic brain signaling pathways. In addition, QCN attenuates AD neuropathology by inhibiting GSK3β and PTEN signaling pathways.

However, QCN has poor bioavailability to cross the BBB. Therefore, different approaches were introduced to enhance QCN bioavailability and to enhance its BBB penetration. Thus, QCN nanoemulsion has higher bioavailability and can cross the BBB. In addition, QCN could an adjuvant therapeutic agent with anti-AD medication to improve the clinical outcomes in AD patients. Hence, future directional clinical trials and prospective studies investigating the potential therapeutic efficacy of QCN alone or in combination with anti-AD medication are recommended.

Collectively, QCN by regulating different signaling pathways could be a promising therapeutic herbal remedy in the management of AD. Further additional studies are recommended in this concern.

## Data Availability

Data sharing is not applicable to this article as no datasets were generated or analyzed during the current study.

## References

[CR1] Abdalla FH, Cardoso AM, Pereira LB, Schmatz R, Gonçalves JF, Stefanello N, Fiorenza AM, Gutierres JM, da Silva Serres JD, Zanini D, Pimentel VC, Vieira JM, Schetinger MRC, Morsch VM, Mazzanti CM (2013) Neuroprotective effect of quercetin in ectoenzymes and acetylcholinesterase activities in cerebral cortex synaptosomes of cadmium-exposed rats. Mol Cell Biochem 381(1–2):1–8. 10.1007/s11010-013-1659-x 23797318 10.1007/s11010-013-1659-x

[CR2] Abdalla FH, Schmatz R, Cardoso AM, Carvalho FB, Baldissarelli J, de Oliveira JS, Rosa MM, Gonçalves Nunes MA, Rubin MA, da Cruz IBM, Barbisan F, Dressler VL, Pereira LB, Schetinger MRC, Morsch VM, Gonçalves JF, Mazzanti CM (2014) Quercetin protects the impairment of memory and anxiogenic-like behavior in rats exposed to cadmium: possible involvement of the acetylcholinesterase and Na+, K+-ATPase activities. Physiol Behav 135:152–167. 10.1016/j.physbeh.2014.06.008 24952260 10.1016/j.physbeh.2014.06.008

[CR3] Adeoluwa OA, Olayinka JN, Adeoluwa GO, Akinluyi ET, Adeniyi FR, Fafure A, Nebo K, Edem EE, Eduviere AT, Abubakar B (2023) Quercetin abrogates lipopolysaccharide-induced depressive-like symptoms by inhibiting neuroinflammation via microglial NLRP3/NFκB/iNOS signaling pathway. Behav Brain Res 450:114503. 10.1016/j.bbr.2023.114503 37209878 10.1016/j.bbr.2023.114503

[CR4] Aggarwal A, Sharma N, Khera A, Sandhir R, Rishi V (2020) Quercetin alleviates cognitive decline in ovariectomized mice by potentially modulating histone acetylation homeostasis. J Nutr Biochem 84:108439. 10.1016/j.jnutbio.2020.108439 32622308 10.1016/j.jnutbio.2020.108439

[CR5] Ahmadi L, Eskandari N, Ghanadian M, Rahmati M, Kasiri N, Etamadifar M, Toghyani M, Alsahebfosoul F (2023) The immunomodulatory aspect of quercetin penta acetate on Th17 cells proliferation and gene expression in multiple sclerosis. Cell J 25(2):110–117. 10.22074/CELLJ.2022.557560.1073 36840457 10.22074/cellj.2022.557560.1073 PMC9968368

[CR6] Ahmed T, Van der Jeugd A, Caillierez R, Buée L, Blum D, D’Hooge R, Balschun D (2020) Chronic sodium selenate treatment restores deficits in cognition and synaptic plasticity in a murine model of tauopathy. Front Mole Neurosci. 10.3389/fnmol.2020.570223 10.3389/fnmol.2020.570223 PMC7578417 33132838

[CR7] Akhtar A, Sah SP (2020) Insulin signaling pathway and related molecules: role in neurodegeneration and Alzheimer’s disease. Neurochem Int 1(135):104707 10.1016/j.neuint.2020.104707 32092326

[CR8] Al-Kuraishy HM, Al-Fakhrany OM, Elekhnawy E, Al-Gareeb AI, Alorabi M, De Waard M, Albogami SM, Batiha GE (2022) Traditional herbs against COVID-19: back to old weapons to combat the new pandemic. Eur J Med Res 27(1):186 36154838 10.1186/s40001-022-00818-5 PMC9510171

[CR9] Al-Kuraishy HM, Al-Gareeb AI, Alsayegh AA, Hakami ZH, Khamjan NA, Saad HM, Batiha GE-S, De Waard M (2023) a potential link between visceral obesity and risk of Alzheimer’s disease. Neurochem Res 48(3):745–766. 10.1007/s11064-022-03817-4 36409447 10.1007/s11064-022-03817-4

[CR10] Al-kuraishy HM, Jabir MS, Albuhadily AK, Al-Gareeb AI, Rafeeq MF (2023a) The link between metabolic syndrome and Alzheimer disease: a mutual relationship and long rigorous investigation. Ageing Res Rev 91:102084. 10.1016/j.arr.2023.102084 37802319 10.1016/j.arr.2023.102084

[CR11] Al-kuraishy HM, Jabir MS, Al-Gareeb AI, Albuhadily AK (2023b) The conceivable role of prolactin hormone in Parkinson disease: the same goal but with different ways. Ageing Res Rev 91:102075. 10.1016/j.arr.2023.102075 37714384 10.1016/j.arr.2023.102075

[CR12] Al-kuraishy HM, Fahad EH, Al-Windy S, El-Sherbeni SA, Negm WA, Batiha GE-S (2024a) The effects of cholesterol and statins on Parkinson’s neuropathology: a narrative review. Inflammopharmacology 32(2):917–925. 10.1007/s10787-023-01400-z 38499742 10.1007/s10787-023-01400-z

[CR13] Al-kuraishy HM, Jabir MS, Al-Gareeb AI, Saad HM, Batiha GE-S, Klionsky DJ (2024b) The beneficial role of autophagy in multiple sclerosis: yes or no? Autophagy 20(2):259–274. 10.1080/15548627.2023.2259281 37712858 10.1080/15548627.2023.2259281 PMC10813579

[CR14] Al-kuraishy HM, Jabir MS, Sulaiman GM, Mohammed HA, Al-Gareeb AI, Albuhadily AK, Jawad SF, Swelum AA, Abomughaid MM (2024c) The role of statins in amyotrophic lateral sclerosis: protective or not? Front Neurosci. 10.3389/fnins.2024.1422912 38903602 10.3389/fnins.2024.1422912 PMC11188367

[CR15] Alaqeel NK, AlSheikh MH, Al-Hariri MT (2022) Quercetin nanoemulsion ameliorates neuronal dysfunction in experimental alzheimer’s disease model. Antioxidants 11(10):1986. 10.3390/antiox11101986 36290710 10.3390/antiox11101986 PMC9598210

[CR16] Alber J, Maruff P, Santos CY, Ott BR, Salloway SP, Yoo DC, Noto RB, Thompson LI, Goldfarb D, Arthur E, Song A, Snyder PJ (2020) Disruption of cholinergic neurotransmission, within a cognitive challenge paradigm, is indicative of Aβ-related cognitive impairment in preclinical Alzheimer’s disease after a 27-month delay interval. Alzheimer’s Res Ther 12(1):31. 10.1186/s13195-020-00599-1 32209123 10.1186/s13195-020-00599-1 PMC7093953

[CR17] Alexander C, Parsaee A, Vasefi M (2023) Polyherbal and multimodal treatments: kaempferol- and quercetin-rich herbs alleviate symptoms of Alzheimer’s disease. Biology 12(11):1453. 10.3390/biology12111453 37998052 10.3390/biology12111453 PMC10669725

[CR18] Ali NH, Al-Kuraishy HM, Al-Gareeb AI, Alexiou A, Papadakis M, AlAseeri AA, Alruwaili M, Saad HM, Batiha GES (2024a) BDNF/TrkB activators in Parkinson’s disease: a new therapeutic strategy. J Cell Mole Med. 10.1111/jcmm.18368 10.1111/jcmm.18368 PMC11096816 38752280

[CR19] Ali NH, Al-kuraishy HM, Al-Gareeb AI, Alnaaim SA, Saad HM, Batiha GE-S (2024b) The molecular pathway of p75 neurotrophin receptor (p75NTR) in Parkinson’s disease: the way of new inroads. Mol Neurobiol 61(5):2469–2480. 10.1007/s12035-023-03727-8 37897634 10.1007/s12035-023-03727-8

[CR20] Alruwaili M, Al-kuraishy HM, Alexiou A, Papadakis M, AlRashdi BM, Elhussieny O, Saad HM, Batiha GE-S (2023) Pathogenic role of fibrinogen in the neuropathology of multiple sclerosis: a tale of sorrows and fears. Neurochem Res 48(11):3255–3269. 10.1007/s11064-023-03981-1 37442896 10.1007/s11064-023-03981-1 PMC10514123

[CR21] AlRuwaili R, Al-kuraishy HM, Al-Gareeb AI, Ali NH, Alexiou A, Papadakis M, Saad HM, Batiha GE-S (2024) The possible role of brain-derived neurotrophic factor in epilepsy. Neurochem Res 49(3):533–547. 10.1007/s11064-023-04064-x 38006577 10.1007/s11064-023-04064-x PMC10884085

[CR22] Alvarez-Arellano L, Salazar-García M, Corona JC (2020) Neuroprotective effects of quercetin in pediatric neurological diseases. Molecules 25(23):5597. 10.3390/molecules25235597 33260783 10.3390/molecules25235597 PMC7731313

[CR23] Álvarez-Berbel I, Espargaró A, Viayna A, Caballero AB, Busquets MA, Gámez P, Luque FJ, Sabaté R (2022) Three to tango: inhibitory effect of quercetin and apigenin on acetylcholinesterase, amyloid-β aggregation and acetylcholinesterase-amyloid interaction. Pharmaceutics 14(11):2342. 10.3390/pharmaceutics14112342 36365159 10.3390/pharmaceutics14112342 PMC9699245

[CR24] Amanzadeh Jajin E, Esmaeili A, Rahgozar S, Noorbakhshnia M (2021) Quercetin-conjugated superparamagnetic iron oxide nanoparticles protect AlCl3-induced neurotoxicity in a rat model of Alzheimer’s disease via antioxidant genes, APP gene, and miRNA-101. Front Neurosci. 10.3389/fnins.2020.598617 33716639 10.3389/fnins.2020.598617 PMC7947204

[CR25] Ansari MA, Abdul HM, Joshi G, Opii WO, Butterfield DA (2009) Protective effect of quercetin in primary neurons against Aβ(1–42): relevance to Alzheimer’s disease. J Nutr Biochem 20(4):269–275. 10.1016/j.jnutbio.2008.03.002 18602817 10.1016/j.jnutbio.2008.03.002 PMC2737260

[CR26] Ardianto C, Lestari D, Primadani LH, Puspitasari DR, Sumartha INB, Nisak K, Budiatin AS, Shinta DW, Andarsari MR, Ifadotunnikmah F, Abdullah ADI, Rahmadi M, Khotib J (2023) Quercetin exerts a protective effect on ischemic stroke-induced memory deficits in mice. J Pharmacol Pharmacother 14(2):133–141. 10.1177/0976500X231189343

[CR27] Balasubramanian R, Bazaz MR, Pasam T, Sharief N, Velip L, Samanthula G, Dandekar MP (2023) Involvement of microbiome gut–brain axis in neuroprotective effect of quercetin in mouse model of repeated mild traumatic brain injury. NeuroMol Med 25(2):242–254 10.1007/s12017-022-08732-z 36481824

[CR28] Basheer N, Smolek T, Hassan I, Liu F, Iqbal K, Zilka N, Novak P (2023) Does modulation of tau hyperphosphorylation represent a reasonable therapeutic strategy for Alzheimer’s disease? From preclinical studies to the clinical trials. Mol Psychiatry 28(6):2197–2214. 10.1038/s41380-023-02113-z 37264120 10.1038/s41380-023-02113-z PMC10611587

[CR29] Batiha GE-S, Beshbishy AM, Ikram M, Mulla ZS, El-Hack MEA, Taha AE, Algammal AM, Elewa YHA (2020) The pharmacological activity, biochemical properties, and pharmacokinetics of the major natural polyphenolic flavonoid: quercetin. Foods 9(3):374. 10.3390/foods9030374 32210182 10.3390/foods9030374 PMC7143931

[CR30] Bayazid AB, Lim BO (2022) Quercetin is an active agent in berries against neurodegenerative diseases progression through modulation of Nrf2/HO1. Nutrients 14(23):5132. 10.3390/nu14235132 36501161 10.3390/nu14235132 PMC9737775

[CR31] Ben-Shachar M, Rozenberg K, Skalka N, Wollman A, Michlin M, Rosenzweig T (2019) Activation of insulin signaling in adipocytes and myotubes by sarcopoterium spinosum extract. Nutrients 11(6):1396. 10.3390/nu11061396 31234331 10.3390/nu11061396 PMC6628217

[CR32] Benetatos J, Bennett RE, Evans HT, Ellis SA, Hyman BT, Bodea L-G, Götz J (2020) PTEN activation contributes to neuronal and synaptic engulfment by microglia in tauopathy. Acta Neuropathol 140(1):7–24. 10.1007/s00401-020-02151-9 32236736 10.1007/s00401-020-02151-9 PMC7300099

[CR33] Bhatia S, Singh M, Singh T, Singh V (2023) Scrutinizing the therapeutic potential of PROTACs in the management of Alzheimer’s disease. Neurochem Res 48(1):13–25 35987974 10.1007/s11064-022-03722-w

[CR34] Butterfield DA (2014) The 2013 SFRBM discovery award: selected discoveries from the butterfield laboratory of oxidative stress and its sequela in brain in cognitive disorders exemplified by Alzheimer disease and chemotherapy induced cognitive impairment. Free Radical Biol Med 74:157–174. 10.1016/j.freeradbiomed.2014.06.006 24996204 10.1016/j.freeradbiomed.2014.06.006 PMC4146642

[CR35] Caccamo A, Maldonado MA, Majumder S, Medina DX, Holbein W, Magrí A, Oddo S (2011) Naturally secreted amyloid-β increases mammalian target of rapamycin (mTOR) activity via a PRAS40-mediated mechanism. J Biol Chem 286(11):8924–8932. 10.1074/jbc.M110.180638 21266573 10.1074/jbc.M110.180638 PMC3058958

[CR36] Caruana M, Cauchi R, Vassallo N (2016) Putative role of red wine polyphenols against brain pathology in Alzheimer’s and Parkinson’s disease. Front Nutrition. 10.3389/fnut.2016.00031 10.3389/fnut.2016.00031 PMC4981604 27570766

[CR37] Chen J, Deng X, Liu N, Li M, Liu B, Fu Q, Qu R, Ma S (2016) Quercetin attenuates tau hyperphosphorylation and improves cognitive disorder via suppression of ER stress in a manner dependent on AMPK pathway. J Funct Foods 22:463–476. 10.1016/j.jff.2016.01.036

[CR38] Chiu Y-J, Teng Y-S, Chen C-M, Sun Y-C, Hsieh-Li HM, Chang K-H, Lee-Chen G-J (2023) A neuroprotective action of quercetin and apigenin through inhibiting aggregation of Aβ and activation of TRKB signaling in a cellular experiment. Biomole Ther 31(3):285–297. 10.4062/biomolther.2022.136 10.4062/biomolther.2022.136 PMC10129860 36646447

[CR39] Choi H, Park H-H, Lee K-Y, Choi N-Y, Yu H-J, Lee YJ, Park J, Huh Y-M, Lee S-H, Koh S-H (2013) Coenzyme Q10 restores amyloid beta-inhibited proliferation of neural stem cells by activating the PI3K pathway. Stem Cells Develop 22(15):2112–2120. 10.1089/scd.2012.0604 10.1089/scd.2012.0604 23509892

[CR40] Dar NJ, Glazner GW (2020) Deciphering the neuroprotective and neurogenic potential of soluble amyloid precursor protein alpha (sAPPα). Cell Mol Life Sci 77(12):2315–2330. 10.1007/s00018-019-03404-x 31960113 10.1007/s00018-019-03404-x PMC11105086

[CR41] Doifode T, Giridharan VV, Generoso JS, Bhatti G, Collodel A, Schulz PE, Forlenza OV, Barichello T (2021) The impact of the microbiota-gut-brain axis on Alzheimer’s disease pathophysiology. Pharmacol Res 164:105314 33246175 10.1016/j.phrs.2020.105314

[CR42] Dvořáková M, Sivoňová M, Trebatická J, Škodáček I, Waczuliková I, Muchová J, Ďuračková Z (2006) The effect of polyphenolic extract from pine bark, pycnogenol ® on the level of glutathione in children suffering from attention deficit hyperactivity disorder (ADHD). Redox Rep 11(4):163–172. 10.1179/135100006X116664 16984739 10.1179/135100006X116664

[CR43] Elfiky AM, Mahmoud AA, Elreedy HA, Ibrahim KS, Ghazy MA (2021) Quercetin stimulates the non-amyloidogenic pathway via activation of ADAM10 and ADAM17 gene expression in aluminum chloride-induced Alzheimer’s disease rat model. Life Sci 285:119964. 10.1016/j.lfs.2021.119964 34537230 10.1016/j.lfs.2021.119964

[CR44] Fan X, Zhao Z, Wang D, Xiao J (2020) Glycogen synthase kinase-3 as a key regulator of cognitive function. Acta Biochim Biophys Sin 52(3):219–230. 10.1093/abbs/gmz156 32147679 10.1093/abbs/gmz156

[CR45] Fan Y, Wang J, He N, Feng H (2021) PLK2 protects retinal ganglion cells from oxidative stress by potentiating Nrf2 signaling via GSK-3β. J Biochem Mole Toxicol. 10.1002/jbt.22815 10.1002/jbt.22815 34047419

[CR46] Forlenza OV, Torres CA, Talib LL, de Paula VJ, Joaquim HPG, Diniz BS, Gattaz WF (2011) Increased platelet GSK3B activity in patients with mild cognitive impairment and Alzheimer’s disease. J Psychiatr Res 45(2):220–224. 10.1016/j.jpsychires.2010.06.002 20576277 10.1016/j.jpsychires.2010.06.002

[CR47] Fraser MM, Bayazitov IT, Zakharenko SS, Baker SJ (2008) Phosphatase and tensin homolog, deleted on chromosome 10 deficiency in brain causes defects in synaptic structure, transmission and plasticity, and myelination abnormalities. Neuroscience 151(2):476–488. 10.1016/j.neuroscience.2007.10.048 18082964 10.1016/j.neuroscience.2007.10.048 PMC2278004

[CR48] Fronza MG, Alves D, Praticò D, Savegnago L (2023) The neurobiology and therapeutic potential of multi-targeting β-secretase, glycogen synthase kinase 3β and acetylcholinesterase in Alzheimer’s disease. Ageing Res Rev 90:102033. 10.1016/j.arr.2023.102033 37595640 10.1016/j.arr.2023.102033

[CR49] Fürst R, Zündorf I (2014) Plant-derived anti-inflammatory compounds: hopes and disappointments regarding the translation of preclinical knowledge into clinical progress. Mediators Inflamm 2014:146832 24987194 10.1155/2014/146832 PMC4060065

[CR50] Gadhave K, Kumar D, Uversky VN, Giri R (2021) A multitude of signaling pathways associated with Alzheimer’s disease and their roles in AD pathogenesis and therapy. Med Res Rev 41(5):2689–2745. 10.1002/med.21719 32783388 10.1002/med.21719 PMC7876169

[CR51] Girotra P, Behl T, Sehgal A, Singh S, Bungau S (2022) Investigation of the molecular role of brain-derived neurotrophic factor in Alzheimer’s disease. J Mole Neurosci. 10.1007/s12031-021-01824-8 10.1007/s12031-021-01824-8 34424488

[CR52] Gizak A, Duda P, Pielka E, McCubrey JA, Rakus D (2020) GSK3 and miRNA in neural tissue: From brain development to neurodegenerative diseases. Biochimica Et Biophysica Acta (BBA)—Mole Cell Res 1867(7):118696. 10.1016/j.bbamcr.2020.118696 10.1016/j.bbamcr.2020.118696 32165184

[CR53] Goiran T, Duplan E, Chami M, Bourgeois A, El Manaa W, Rouland L, Dunys J, Lauritzen I, You H, Stambolic V, Biféri M-G, Barkats M, Pimplikar SW, Sergeant N, Colin M, Morais VA, Pardossi-Piquard R, Checler F, Alves da Costa C (2018) β-Amyloid precursor protein intracellular domain controls mitochondrial function by modulating phosphatase and tensin homolog-induced kinase 1 transcription in cells and in Alzheimer mice models. Biol Psychiat 83(5):416–427. 10.1016/j.biopsych.2017.04.011 28587718 10.1016/j.biopsych.2017.04.011

[CR54] Grande V, Manassero G, Vercelli A (2014) Neuroprotective and anti-inflammatory roles of the phosphatase and tensin homolog deleted on chromosome ten (PTEN) inhibition in a mouse model of temporal lobe epilepsy. PLoS ONE 9(12):e114554. 10.1371/journal.pone.0114554 25501575 10.1371/journal.pone.0114554 PMC4264755

[CR55] Grewal AK, Singh TG, Sharma D, Sharma V, Singh M, Rahman MdH, Najda A, Walasek-Janusz M, Kamel M, Albadrani GM, Akhtar MF, Saleem A, Abdel-Daim MM (2021) Mechanistic insights and perspectives involved in neuroprotective action of quercetin. Biomed Pharmacother 140:111729. 10.1016/j.biopha.2021.111729 34044274 10.1016/j.biopha.2021.111729

[CR56] Guo C, Wang W-J, Liao Y-C, Zhao C, Yin Y, Yao M-N, Ding Y, Wang J-W (2022) Effect and mechanisms of quercetin for experimental focal cerebral ischemia: a systematic review and meta-analysis. Oxid Med Cell Longev 2022:1–13. 10.1155/2022/9749461 10.1155/2022/9749461 PMC8896934 35251482

[CR57] Gupta S, Singh V, Ganesh S, Singhal NK, Sandhir R (2022) siRNA Mediated GSK3β knockdown targets insulin signaling pathway and rescues Alzheimer’s disease pathology: evidence from *in vitro* and *in vivo* studies. ACS Appl Mater Interfaces 14(1):69–93. 10.1021/acsami.1c15305 34967205 10.1021/acsami.1c15305

[CR58] Hao Q, Henning SM, Magyar CE, Said J, Zhong J, Rettig MB, Vadgama JV, Wang P (2024) Enhanced chemoprevention of prostate cancer by combining arctigenin with green tea and quercetin in prostate-specific phosphatase and tensin homolog knockout mice. Biomolecules 14(1):105. 10.3390/biom14010105 38254705 10.3390/biom14010105 PMC10813217

[CR59] He Z, Li X, Han S, Ren B, Hu X, Li N, Du X, Ni J, Yang X, Liu Q (2021) Bis(ethylmaltolato)oxidovanadium (IV) attenuates amyloid-beta-mediated neuroinflammation by inhibiting NF-κB signaling pathway via a PPARγ-dependent mechanism. Metallomics. 10.1093/mtomcs/mfab036 34124763 10.1093/mtomcs/mfab036

[CR60] Ho C-L, Kao N-J, Lin C-I, Cross T-WL, Lin S-H (2022) Quercetin increases mitochondrial biogenesis and reduces free radicals in neuronal SH-SY5Y Cells. Nutrients 14(16):3310. 10.3390/nu14163310 36014814 10.3390/nu14163310 PMC9414536

[CR61] Jaisamut P, Wanna S, Limsuwan S, Chusri S, Wiwattanawongsa K, Wiwattanapatapee R (2021) Enhanced oral bioavailability and improved biological activities of a quercetin/resveratrol combination using a liquid self-microemulsifying drug delivery system. Planta Med 87(04):336–346. 10.1055/a-1270-7606 33176379 10.1055/a-1270-7606

[CR62] Javanbakht P, Yazdi FR, Taghizadeh F, Khadivi F, Hamidabadi HG, Kashani IR, Zarini D, Mojaverrostami S (2023) Quercetin as a possible complementary therapy in multiple sclerosis: anti-oxidative, anti-inflammatory and remyelination potential properties. Heliyon 9(11):e21741. 10.1016/j.heliyon.2023.e21741 37954351 10.1016/j.heliyon.2023.e21741 PMC10638059

[CR63] Jellinger KA (2020) Pathobiological subtypes of Alzheimer disease. Dement Geriatr Cogn Disord 49(4):321–333. 10.1159/000508625 33429401 10.1159/000508625

[CR64] Jiang W, Luo T, Li S, Zhou Y, Shen X-Y, He F, Xu J, Wang H-Q (2016) Quercetin protects against okadaic acid-induced injury via MAPK and PI3K/Akt/GSK3β Signaling pathways in HT22 hippocampal neurons. PLoS ONE 11(4):e0152371. 10.1371/journal.pone.0152371 27050422 10.1371/journal.pone.0152371 PMC4822954

[CR65] Jiang M, Zhang X, Yan X, Mizutani S, Kashiwazaki H, Ni J, Wu Z (2021) GSK3β is involved in promoting Alzheimer’s disease pathologies following chronic systemic exposure to porphyromonas gingivalis lipopolysaccharide in amyloid precursor proteinNL-F/NL-F knock-in mice. Brain Behav Immun 98:1–12. 10.1016/j.bbi.2021.08.213 34391814 10.1016/j.bbi.2021.08.012 PMC8849844

[CR66] Jin T, Zhang Y, Botchway BOA, Huang M, Lu Q, Liu X (2023) Quercetin activates the sestrin2/AMPK/SIRT1 axis to improve amyotrophic lateral sclerosis. Biomed Pharmacother 161:114515. 10.1016/j.biopha.2023.114515 36913894 10.1016/j.biopha.2023.114515

[CR67] Jurcău MC, Andronie-Cioara FL, Jurcău A, Marcu F, Ţiț DM, Pașcalău N, Nistor-Cseppentö DC (2022) The link between oxidative stress, mitochondrial dysfunction and neuroinflammation in the pathophysiology of Alzheimer’s disease: therapeutic implications and future perspectives. Antioxidants 11(11):2167. 10.3390/antiox11112167 36358538 10.3390/antiox11112167 PMC9686795

[CR68] Kalra P, Garg C, Singh V, Singh TG, Grewal AK. Neuroprotection induced by quercetin. In Natural Molecules in Neuroprotection and Neurotoxicity 2024 (pp. 1757–1783). Academic Press

[CR69] Khan A, Ali T, Rehman SU, Khan MS, Alam SI, Ikram M, Muhammad T, Saeed K, Badshah H, Kim MO (2018) Neuroprotective effect of quercetin against the detrimental effects of LPS in the adult mouse brain. Front Pharmacol. 10.3389/fphar.2018.01383 30618732 10.3389/fphar.2018.01383 PMC6297180

[CR70] Khan H, Ullah H, Aschner M, Cheang WS, Akkol EK (2019) Neuroprotective effects of quercetin in Alzheimer’s disease. Biomolecules 10(1):59. 10.3390/biom10010059 31905923 10.3390/biom10010059 PMC7023116

[CR71] Kim J, Lee HJ, Lee KW (2010) Naturally occurring phytochemicals for the prevention of Alzheimer’s disease. J Neurochem 112(6):1415–1430. 10.1111/j.1471-4159.2009.06562.x 20050972 10.1111/j.1471-4159.2009.06562.x

[CR72] Kumar M, Bansal N (2022) Implications of phosphoinositide 3-kinase-akt (PI3K-Akt) pathway in the pathogenesis of Alzheimer’s disease. Mol Neurobiol 59(1):354–385. 10.1007/s12035-021-02611-7 34699027 10.1007/s12035-021-02611-7

[CR73] Kumar S, Modgil S, Bammidi S, Minhas G, Shri R, Kaushik S, Singh V, Anand A (2020) Allium cepa exerts neuroprotective effect on retinal ganglion cells of pterygopalatine artery (PPA) ligated mice. J Ayurveda Integr Med 11(4):489–494 32088091 10.1016/j.jaim.2019.08.002 PMC7772493

[CR74] Lazo-Gomez R, Tapia R (2017) Quercetin prevents spinal motor neuron degeneration induced by chronic excitotoxic stimulus by a sirtuin 1-dependent mechanism. Transl Neurodegener 6(1):31. 10.1186/s40035-017-0102-8 29201361 10.1186/s40035-017-0102-8 PMC5697078

[CR75] Lee GB, Kim Y, Lee KE, Vinayagam R, Singh M, Kang SG (2024) Anti-inflammatory effects of quercetin, rutin, and troxerutin result from the inhibition of NO production and the reduction of COX-2 levels in RAW 264.7 cells treated with LPS. Appl Biochem Biotechnol. 10.1007/s12010-024-05003-4 39096472 10.1007/s12010-024-05003-4

[CR76] Lei X, Chao H, Zhang Z, Lv J, Li S, Wei H, Xue R, Li F, Li Z (2015) Neuroprotective effects of quercetin in a mouse model of brain ischemic/reperfusion injury via anti-apoptotic mechanisms based on the Akt pathway. Mol Med Rep 12(3):3688–3696. 10.3892/mmr.2015.3857 26016839 10.3892/mmr.2015.3857

[CR77] Leroy K, Yilmaz Z, Brion J-P (2007) Increased level of active GSK-3β in Alzheimer’s disease and accumulation in argyrophilic grains and in neurones at different stages of neurofibrillary degeneration. Neuropathol Appl Neurobiol 33(1):43–55. 10.1111/j.1365-2990.2006.00795.x 17239007 10.1111/j.1365-2990.2006.00795.x

[CR78] Li Y, Zhang J, Wan J, Liu A, Sun J (2020) Melatonin regulates Aβ production/clearance balance and Aβ neurotoxicity: a potential therapeutic molecule for Alzheimer’s disease. Biomed Pharmacother 132:110887. 10.1016/j.biopha.2020.110887 33254429 10.1016/j.biopha.2020.110887

[CR79] Li R, Qi J, Yang Y, Wu Y, Yin P, Zhou M, Qian Z, LeBaige MH, McMillin SE, Guo H, Lin H (2021) Disease burden and attributable risk factors of Alzheimer’s disease and dementia in China from 1990 to 2019. J Prevent of Alzheimer’s Dis. 10.14283/jpad.2021.69 10.14283/jpad.2021.69 35543004

[CR80] Liao Y, Mai X, Wu X, Hu X, Luo X, Zhang G (2022) Exploring the Inhibition of quercetin on acetylcholinesterase by multispectroscopic and in silico approaches and evaluation of its neuroprotective effects on PC12 cells. Molecules 27(22):7971. 10.3390/molecules27227971 36432070 10.3390/molecules27227971 PMC9699400

[CR81] Lim YY, Maruff P, Barthélemy NR, Goate A, Hassenstab J, Sato C, Fagan AM, Benzinger TLS, Xiong C, Cruchaga C, Levin J, Farlow MR, Graff-Radford NR, Laske C, Masters CL, Salloway S, Schofield PR, Morris JC, Bateman RJ, Xu X (2022) Association of *BDNF* val66met with tau hyperphosphorylation and cognition in dominantly inherited Alzheimer disease. JAMA Neurol 79(3):261. 10.1001/jamaneurol.2021.5181 35099506 10.1001/jamaneurol.2021.5181 PMC8804973

[CR82] Lin Z-H, Liu Y, Xue N-J, Zheng R, Yan Y-Q, Wang Z-X, Li Y-L, Ying C-Z, Song Z, Tian J, Pu J-L, Zhang B-R (2022) Quercetin protects against MPP+/MPTP-induced dopaminergic neuron death in parkinson’s disease by inhibiting ferroptosis. Oxid Med Cell Longev 2022:1–17. 10.1155/2022/7769355 10.1155/2022/7769355 PMC9467739 36105483

[CR83] Liu Y, Liu F, Grundke-Iqbal I, Iqbal K, Gong C (2011) Deficient brain insulin signalling pathway in Alzheimer’s disease and diabetes. J Pathol 225(1):54–62. 10.1002/path.2912 21598254 10.1002/path.2912 PMC4484598

[CR84] Liu L, Liu Y, Zhen Y, Guo T, Wang C, Shen L, Li W (2022) Quercetin inhibits cytotoxicity of PC12 cells induced by amyloid-beta 25–35 via stimulating estrogen receptor α, activating ERK1/2, and inhibiting apoptosis. Open Life Sci 17(1):230–242. 10.1515/biol-2021-0014

[CR85] Mahadev M, Nandini HS, Ramu R, Gowda DV, Almarhoon ZM, Al-Ghorbani M, Mabkhot YN (2022) Fabrication and evaluation of quercetin nanoemulsion: a delivery system with improved bioavailability and therapeutic efficacy in diabetes mellitus. Pharmaceuticals. 10.3390/ph15010070 35056127 10.3390/ph15010070 PMC8779357

[CR86] Maioli S, Leander K, Nilsson P, Nalvarte I (2021) Estrogen receptors and the aging brain. Essays Biochem 65(6):913–925. 10.1042/EBC20200162 34623401 10.1042/EBC20200162 PMC8628183

[CR87] Mannan A, Singh TG, Singh V, Garg N, Kaur A, Singh M (2022) Insights into the mechanism of the therapeutic potential of herbal monoamine oxidase inhibitors in neurological diseases. Curr Drug Targets 23(3):286–310 34238153 10.2174/1389450122666210707120256

[CR88] Mao L-L, Hao D-L, Mao X-W, Xu Y-F, Huang T-T, Wu B-N, Wang L-H (2015) Neuroprotective effects of bisperoxovanadium on cerebral ischemia by inflammation inhibition. Neurosci Lett 602:120–125. 10.1016/j.neulet.2015.06.040 26141612 10.1016/j.neulet.2015.06.040

[CR89] Megur A, Baltriukienė D, Bukelskienė V, Burokas A (2021) The microbiota–gut–brain axis and Alzheimer’s disease: neuroinflammation is to blame? Nutrients. 10.3390/nu13010037 10.3390/nu13010037 PMC7824474 33374235

[CR90] Miao Z, Miao Z, Wang S, Shi X, Xu S (2021) Quercetin antagonizes imidacloprid-induced mitochondrial apoptosis through PTEN/PI3K/AKT in grass carp hepatocytes. Environ Pollut 290:118036. 10.1016/j.envpol.2021.118036 34488159 10.1016/j.envpol.2021.118036

[CR91] Molaei A, Hatami H, Dehghan G, Sadeghian R, Khajehnasiri N (2020) Synergistic effects of quercetin and regular exercise on the recovery of spatial memory and reduction of parameters of oxidative stress in an animal model of Alzheimer’s disease. EXCLI J 19:596–612. 10.17179/excli2019-2082 32483406 10.17179/excli2019-2082 PMC7257248

[CR92] Mori Y, Tsuji M, Oguchi T, Kasuga K, Kimura A, Futamura A, Sugimoto A, Kasai H, Kuroda T, Yano S, Hieda S, Kiuchi Y, Ikeuchi T, Ono K (2021) Serum BDNF as a potential biomarker of Alzheimer’s disease: verification through assessment of serum, cerebrospinal fluid, and medial temporal lobe atrophy. Front Neurol. 10.3389/fneur.2021.653267 33967943 10.3389/fneur.2021.653267 PMC8102980

[CR93] Muralidar S, Ambi SV, Sekaran S, Thirumalai D, Palaniappan B (2020) Role of tau protein in Alzheimer’s disease: the prime pathological player. Int J Biol Macromol 163:1599–1617. 10.1016/j.ijbiomac.2020.07.327 32784025 10.1016/j.ijbiomac.2020.07.327

[CR94] Naghizadeh M, Mirshekar MA, Montazerifar F, Saadat S, Koushki AS, Maskouni SJ, Afsharfar M, Arabmoazzen S (2021) Effects of quercetin on spatial memory, hippocampal antioxidant defense and BDNF concentration in a rat model of Parkinson’s disease: an electrophysiological study. Avicenna J Phytomed 11(6):599–609. 10.22038/AJP.2021.18526 34804897 10.22038/AJP.2021.18526 PMC8588960

[CR95] Nakagawa T, Ohta K (2019) Quercetin regulates the integrated stress response to improve memory. Int J Mol Sci 20(11):2761. 10.3390/ijms20112761 31195662 10.3390/ijms20112761 PMC6600673

[CR96] Nishihira J, Nishimura M, Kurimoto M, Kagami-Katsuyama H, Hattori H, Nakagawa T, Muro T, Kobori M (2021) The effect of 24-week continuous intake of quercetin-rich onion on age-related cognitive decline in healthy elderly people: a randomized, double-blind, placebo-controlled, parallel-group comparative clinical trial. J Clin Biochem Nutrition 69(2):21–17. 10.3164/jcbn.21-17 10.3164/jcbn.21-17 PMC8482389 34616111

[CR97] Nishimura M, Ohkawara T, Nakagawa T, Muro T, Sato Y, Satoh H, Kobori M, Nishihira J (2017) A randomized, double-blind, placebo-controlled study evaluating the effects of quercetin-rich onion on cognitive function in elderly subjects. Funct Foods Health Dis 7(6):353. 10.31989/ffhd.v7i6.334

[CR98] Paris D, Mathura V, Ait-Ghezala G, Beaulieu-Abdelahad D, Patel N, Bachmeier C, Mullan M (2011) Flavonoids lower Alzheimer’s Aß production via an NFkB dependent mechanism. Bioinformation 6(6):229–236. 10.6026/97320630006229 21738321 10.6026/97320630006229 PMC3124791

[CR99] Park D-J, Kang J-B, Shah M-A, Koh P-O (2019) Quercetin alleviates the injury-induced decrease of protein phosphatase 2A subunit B in cerebral ischemic animal model and glutamate-exposed HT22 cells. J Vet Med Sci 81(7):1047–1054. 10.1292/jvms.19-0094 31092742 10.1292/jvms.19-0094 PMC6656806

[CR100] Pecoraro C, Faggion B, Balboni B, Carbone D, Peters GJ, Diana P, Assaraf YG, Giovannetti E (2021) GSK3β as a novel promising target to overcome chemoresistance in pancreatic cancer. Drug Resist Updates 58:100779. 10.1016/j.drup.2021.100779 10.1016/j.drup.2021.100779 34461526

[CR101] Pfundstein G, Nikonenko AG, Sytnyk V (2022) Amyloid precursor protein (APP) and amyloid β (Aβ) interact with cell adhesion molecules: Implications in Alzheimer’s disease and normal physiology. Front Cell Develop Biol. 10.3389/fcell.2022.969547 10.3389/fcell.2022.969547 PMC9360506 35959488

[CR102] Pires M, Rego AC (2023) Apoe4 and Alzheimer’s disease pathogenesis—mitochondrial deregulation and targeted therapeutic strategies. Int J Mol Sci 24(1):778. 10.3390/ijms24010778 36614219 10.3390/ijms24010778 PMC9821307

[CR103] Pláteník J, Fišar Z, Buchal R, Jirák R, Kitzlerová E, Zvěřová M, Raboch J (2014) GSK3β, CREB, and BDNF in peripheral blood of patients with Alzheimer’s disease and depression. Prog Neuropsychopharmacol Biol Psychiatry 50:83–93. 10.1016/j.pnpbp.2013.12.001 24334212 10.1016/j.pnpbp.2013.12.001

[CR104] Predes D, Maia LA, Matias I, Araujo HPM, Soares C, Barros-Aragão FGQ, Oliveira LFS, Reis RR, Amado NG, Simas ABC, Mendes FA, Gomes FCA, Figueiredo CP, Abreu JG (2022) The flavonol quercitrin hinders GSK3 activity and potentiates the Wnt/β-catenin signaling pathway. Int J Mol Sci 23(20):12078. 10.3390/ijms232012078 36292931 10.3390/ijms232012078 PMC9602613

[CR105] Prossomariti A, Piazzi G, Alquati C, Ricciardiello L (2020) Are Wnt/β-catenin and PI3K/AKT/mTORC1 distinct pathways in colorectal cancer? Cell Mol Gastroenterol Hepatol 10(3):491–506. 10.1016/j.jcmgh.2020.04.007 32334125 10.1016/j.jcmgh.2020.04.007 PMC7369353

[CR106] Qureshi AA, Tan X, Reis JC, Badr MZ, Papasian CJ, Morrison DC, Qureshi N (2011) Inhibition of nitric oxide in LPS-stimulated macrophages of young and senescent mice by δ-tocotrienol and quercetin. Lipids Health Dis 10(1):239. 10.1186/1476-511X-10-239 22185406 10.1186/1476-511X-10-239 PMC3267680

[CR107] Rahman SO, Khan T, Iqubal A, Agarwal S, Akhtar M, Parvez S, Shah ZA, Najmi AK (2023) Association between insulin and Nrf2 signalling pathway in Alzheimer’s disease: a molecular landscape. Life Sci 328:121899 37394097 10.1016/j.lfs.2023.121899

[CR108] Randhawa K, Singh V, Kaur S, Kaur R, Kumar S, Shri R (2021) Isolation of pleurotus florida derived acetylcholinesterase inhibitor for the treatment of cognitive dysfunction in mice. Food Sci Human Wellness 10(4):490–496

[CR109] Reddy VP, Aryal P, Robinson S, Rafiu R, Obrenovich M, Perry G (2020) Polyphenols in Alzheimer’s disease and in the gut–brain axis. Microorganisms 8(2):199 32023969 10.3390/microorganisms8020199 PMC7074796

[CR110] Riis S, Murray JB, O’Connor R (2020) IGF-1 signalling regulates mitochondria dynamics and turnover through a conserved GSK-3β–Nrf2–BNIP3 pathway. Cells 9(1):147. 10.3390/cells9010147 31936236 10.3390/cells9010147 PMC7016769

[CR111] Russo, G. L., Russo, M., Spagnuolo, C., Tedesco, I., Bilotto, S., Iannitti, R., & Palumbo, R. (2014). *Quercetin: A Pleiotropic Kinase Inhibitor Against Cancer* (pp. 185–205). 10.1007/978-3-642-38007-5_11 10.1007/978-3-642-38007-5_11 24114481

[CR112] Sabogal-Guáqueta AM, Muñoz-Manco JI, Ramírez-Pineda JR, Lamprea-Rodriguez M, Osorio E, Cardona-Gómez GP (2015) The flavonoid quercetin ameliorates Alzheimer’s disease pathology and protects cognitive and emotional function in aged triple transgenic Alzheimer’s disease model mice. Neuropharmacology 93:134–145. 10.1016/j.neuropharm.2015.01.027 25666032 10.1016/j.neuropharm.2015.01.027 PMC4387064

[CR113] Sato M, Murakami K, Uno M, Nakagawa Y, Katayama S, Akagi K, Masuda Y, Takegoshi K, Irie K (2013) Site-specific inhibitory mechanism for amyloid β42 aggregation by catechol-type flavonoids targeting the lys residues. J Biol Chem 288(32):23212–23224. 10.1074/jbc.M113.464222 23792961 10.1074/jbc.M113.464222 PMC3743493

[CR114] Scheltens P, De Strooper B, Kivipelto M, Holstege H, Chételat G, Teunissen CE, Cummings J, van der Flier WM (2021) Alzheimer’s disease. Lancet 397(10284):1577–1590. 10.1016/S0140-6736(20)32205-4 33667416 10.1016/S0140-6736(20)32205-4 PMC8354300

[CR115] Sharifi-Rad M, Lankatillake C, Dias DA, Docea AO, Mahomoodally MF, Lobine D, Chazot PL, Kurt B, Boyunegmez Tumer T, Catarina Moreira A, Sharopov F, Martorell M, Martins N, Cho WC, Calina D, Sharifi-Rad J (2020) Impact of natural Compounds on neurodegenerative disorders: from preclinical to pharmacotherapeutics. J Clin Med 9(4):1061. 10.3390/jcm9041061 32276438 10.3390/jcm9041061 PMC7231062

[CR116] Shen H, Wang J, Shen L, Wang H, Li W, Ding X (2020) Phosphatase and tensin homolog deletion enhances neurite outgrowth during neural stem cell differentiation. Neuropathology 40(3):224–231. 10.1111/neup.12633 32037610 10.1111/neup.12633

[CR117] Singh NK, Garabadu D (2021) Quercetin exhibits α7nAChR/Nrf2/HO-1-mediated neuroprotection against STZ-induced mitochondrial toxicity and cognitive impairments in experimental rodents. Neurotox Res 39(6):1859–1879. 10.1007/s12640-021-00410-5 34554409 10.1007/s12640-021-00410-5

[CR118] Singh V, Chauhan G, Shri R (2021) Anti-depressant like effects of quercetin 4’-O-glucoside from *Allium cepa via* regulation of brain oxidative stress and monoamine levels in mice subjected to unpredictable chronic mild stress. Nutr Neurosci 24(1):35–44 31368414 10.1080/1028415X.2019.1587247

[CR119] Singh P, Arif Y, Bajguz A, Hayat S (2021) The role of quercetin in plants. Plant Physiol Biochem 166:10–19. 10.1016/j.plaphy.2021.05.023 34087741 10.1016/j.plaphy.2021.05.023

[CR121] Song Y, Li S, Li X, Chen X, Wei Z, Liu Q, Cheng Y (2020) The effect of estrogen replacement therapy on Alzheimer’s disease and Parkinson’s disease in postmenopausal women: a meta-analysis. Front Neurosci. 10.3389/fnins.2020.00157 32210745 10.3389/fnins.2020.00157 PMC7076111

[CR122] Song L, Oseid DE, Wells EA, Robinson AS (2022) The Interplay between GSK3β and tau Ser262 phosphorylation during the progression of tau pathology. Int J Mol Sci 23(19):11610. 10.3390/ijms231911610 36232909 10.3390/ijms231911610 PMC9569960

[CR123] Sonoda Y, Mukai H, Matsuo K, Takahashi M, Ono Y, Maeda K, Akiyama H, Kawamata T (2010) Accumulation of tumor-suppressor PTEN in Alzheimer neurofibrillary tangles. Neurosci Lett 471(1):20–24. 10.1016/j.neulet.2009.12.078 20056128 10.1016/j.neulet.2009.12.078

[CR124] Sultzer DL, Lim AC, Gordon HL, Yarns BC, Melrose RJ (2022) Cholinergic receptor binding in unimpaired older adults, mild cognitive impairment, and Alzheimer’s disease dementia. Alzheimer’s Res Ther 14(1):25. 10.1186/s13195-021-00954-w 35130968 10.1186/s13195-021-00954-w PMC8819935

[CR125] Sun P, Yang Y, Yang L, Qian Y, Liang M, Chen H, Zhang J, Qiu Y, Guo L, Fu S (2024) Quercetin protects blood-brain barrier integrity via the PI3K/Akt/Erk signaling pathway in a mouse model of meningitis induced by glaesserella parasuis. Biomolecules. 10.3390/biom14060696 38927100 10.3390/biom14060696 PMC11201931

[CR126] Talbot K, Wang HY, Kazi H, Han LY, Bakshi KP, Stucky A, Fuino RL, Kawaguchi KR, Samoyedny AJ, Wilson RS, Arvanitakis Z, Schneider JA, Wolf BA, Bennett DA, Trojanowski JQ, Arnold SE (2012) Demonstrated brain insulin resistance in Alzheimer’s disease patients is associated with IGF-1 resistance, IRS-1 dysregulation, and cognitive decline. J Clin Investig 122(4):1316–1338. 10.1172/JCI59903 22476197 10.1172/JCI59903 PMC3314463

[CR127] Tamtaji OR, Hadinezhad T, Fallah M, Shahmirzadi AR, Taghizadeh M, Behnam M, Asemi Z (2020) The therapeutic potential of quercetin in Parkinson’s disease: insights into its molecular and cellular regulation. Curr Drug Targets 21(5):509–518. 10.2174/1389450120666191112155654 31721700 10.2174/1389450120666191112155654

[CR128] Tan Z, Yang G, Qiu J, Yan W, Liu Y, Ma Z, Li J, Liu J, Shan N (2022) Quercetin alleviates demyelination through regulating microglial phenotype transformation to mitigate neuropsychiatric symptoms in mice with vascular dementia. Mol Neurobiol 59(5):3140–3158. 10.1007/s12035-021-02712-3 35267135 10.1007/s12035-021-02712-3

[CR129] Tramutola A, Triplett JC, Di Domenico F, Niedowicz DM, Murphy MP, Coccia R, Perluigi M, Butterfield DA (2015) Alteration of mTOR signaling occurs early in the progression of Alzheimer disease (AD): analysis of brain from subjects with pre-clinical AD, amnestic mild cognitive impairment and late-stage AD. J Neurochem 133(5):739–749. 10.1111/jnc.13037 25645581 10.1111/jnc.13037

[CR130] Trejo-Lopez JA, Yachnis AT, Prokop S (2022) Neuropathology of Alzheimer’s disease. Neurotherapeutics 19(1):173–185. 10.1007/s13311-021-01146-y 34729690 10.1007/s13311-021-01146-y PMC9130398

[CR131] Turkistani A, Al-kuraishy HM, Al-Gareeb AI, Albuhadily AK, Elhussieny O, Al-Farga A, Aqlan F, Saad HM, Batiha GES (2024) The functional and molecular roles of p75 neurotrophin receptor (p75NTR) in epilepsy. J Central Nerv Syst Dis. 10.1177/11795735241247810 10.1177/11795735241247810 PMC11036928 38655152

[CR132] Uddin MdS, Hasana S, Ahmad J, Hossain MdF, Rahman MdM, Behl T, Rauf A, Ahmad A, Hafeez A, Perveen A, Ashraf GM (2021) Anti-neuroinflammatory potential of polyphenols by inhibiting NF-κB to halt Alzheimer’s disease. Curr Pharm des 27(3):402–414. 10.2174/1381612826666201118092422 33213314 10.2174/1381612826666201118092422

[CR133] Wang G, Li Y, Lei C, Lei X, Zhu X, Yang L, Zhang R (2021a) Quercetin exerts antidepressant and cardioprotective effects in estrogen receptor α-deficient female mice via BDNF-AKT/ERK1/2 signaling. J Steroid Biochem Mol Biol 206:105795. 10.1016/j.jsbmb.2020.105795 33246157 10.1016/j.jsbmb.2020.105795

[CR134] Wang, Q., Wang, J., Xiang, H., Ding, P., Wu, T., & Ji, G. (2021). The biochemical and clinical implications of phosphatase and tensin homolog deleted on chromosome ten in different cancers. *Am J Cancer Res,* 11(12). www.ajcr.us/PMC8727805 35018228

[CR135] Wang W-W, Han R, He H-J, Li J, Chen S-Y, Gu Y, Xie C (2021c) Administration of quercetin improves mitochondria quality control and protects the neurons in 6-OHDA-lesioned Parkinson’s disease models. Aging 13(8):11738–11751. 10.18632/aging.202868 33878030 10.18632/aging.202868 PMC8109056

[CR136] Wang G, Wang Y, Yao L, Gu W, Zhao S, Shen Z, Lin Z, Liu W, Yan T (2022a) Pharmacological activity of quercetin: an updated review. Evidence-Based Complement Alternat Med 2022:1–12. 10.1155/2022/3997190 10.1155/2022/3997190 PMC9731755 36506811

[CR137] Wang T, Chen Z, Chen H, Yu X, Wang L, Liu X (2022b) Brusatol inhibits the growth of renal cell carcinoma by regulating the PTEN/PI3K/AKT pathway. J Ethnopharmacol 288:115020. 10.1016/j.jep.2022.115020 35066068 10.1016/j.jep.2022.115020

[CR138] Wang X, Feng S, Deng Q, Wu C, Duan R, Yang L (2024) The role of estrogen in Alzheimer’s disease pathogenesis and therapeutic potential in women. Mol Cell Biochem. 10.1007/s11010-024-05071-4 39088186 10.1007/s11010-024-05071-4

[CR139] Wei H, Zhang H, Wang X, Xie J, An D, Wan L, Wang J, Zeng Y, Shu X, Westermarck J, Lu Y, Ohlmeyer M, Liu R (2020) Direct activation of protein phosphatase 2A (PP2A) by tricyclic sulfonamides ameliorates Alzheimer’s disease pathogenesis in cell and animal models. Neurotherapeutics 17(3):1087–1103. 10.1007/s13311-020-00841-6 32096091 10.1007/s13311-020-00841-6 PMC7609734

[CR140] Xu Y, Wei L, Tang S, Shi Q, Wu B, Yang X, Zou Y, Wang X, Ao Q, Meng L, Wei X, Zhang N, Li Y, Lan C, Chen M, Li X, Lu C (2021) Regulation PP2Ac methylation ameliorating autophagy dysfunction caused by Mn is associated with mTORC1/ULK1 pathway. Food Chem Toxicol 156:112441. 10.1016/j.fct.2021.112441 34363881 10.1016/j.fct.2021.112441

[CR141] Yang D, Wang T, Long M, Li P (2020) Quercetin: its main pharmacological activity and potential application in clinical medicine. Oxid Med Cell Longev 2020:1–13. 10.1155/2020/8825387 10.1155/2020/8825387 PMC7790550 33488935

[CR142] Yang J, Liu Y, Lu S, Sun X, Yin Y, Wang K, Liu S (2022) Coix seed oil regulates mitochondrial functional damage to induce apoptosis of human pancreatic cancer cells via the PTEN/PI3K/AKT signaling pathway. Mol Biol Rep 49(7):5897–5909. 10.1007/s11033-022-07371-8 35543827 10.1007/s11033-022-07371-8

[CR143] Yao R-Q, Qi D-S, Yu H-L, Liu J, Yang L-H, Wu X-X (2012) Quercetin attenuates cell apoptosis in focal cerebral ischemia rat brain via activation of BDNF–TrkB–PI3K/Akt signaling pathway. Neurochem Res 37(12):2777–2786. 10.1007/s11064-012-0871-5 22936120 10.1007/s11064-012-0871-5

[CR144] Yiannopoulou KG, Papageorgiou SG (2020) Current and future treatments in Alzheimer disease: an update. J Central Nerv Syst Dis 12:117957352090739. 10.1177/1179573520907397 10.1177/1179573520907397 PMC7050025 32165850

[CR145] Yu X, Li Y, Mu X (2020) Effect of quercetin on PC12 Alzheimer’s disease cell model induced by A *β*_25–35_ and its mechanism based on sirtuin1/Nrf2/HO-1 pathway. Biomed Res Int 2020:1–10. 10.1155/2020/8210578 10.1155/2020/8210578 PMC7201675 32420373

[CR146] Zaplatic E, Bule M, Shah SZA, Uddin MdS, Niaz K (2019) Molecular mechanisms underlying protective role of quercetin in attenuating Alzheimer’s disease. Life Sci 224:109–119. 10.1016/j.lfs.2019.03.055 30914316 10.1016/j.lfs.2019.03.055

[CR147] Zarneshan SN, Fakhri S, Khan H (2022) Targeting Akt/CREB/BDNF signaling pathway by ginsenosides in neurodegenerative diseases: a mechanistic approach. Pharmacol Res 177:106099. 10.1016/j.phrs.2022.106099 35092819 10.1016/j.phrs.2022.106099

[CR148] Zhang H, Wei W, Zhao M, Ma L, Jiang X, Pei H, Cao Y, Li H (2021) Interaction between Aβ and tau in the pathogenesis of Alzheimer’s disease. Int J Biol Sci 17(9):2181–2192. 10.7150/ijbs.57078 34239348 10.7150/ijbs.57078 PMC8241728

[CR149] Zheng M, Wang P (2021) Role of insulin receptor substance-1 modulating PI3K/Akt insulin signaling pathway in Alzheimer’s disease. 3 Biotech 11(4):179. 10.1007/s13205-021-02738-3 33927970 10.1007/s13205-021-02738-3 PMC7981362

[CR150] Zheng L, Zhu H-Z, Wang B-T, Zhao Q-H, Du X-B, Zheng Y, Jiang L, Ni J-Z, Zhang Y, Liu Q (2016) Sodium selenate regulates the brain ionome in a transgenic mouse model of Alzheimer’s disease. Sci Rep 6(1):39290. 10.1038/srep39290 28008954 10.1038/srep39290 PMC5180247

[CR151] Zhou Y, Yang D, Chen H, Zheng C, Jiang H, Liu X, Huang X, Ye S, Song S, Jiang N, Zhao Z, Ma S, Ma J, Huang K, Chen C, Fan X, Gong Y, Wang X, Fan J, Shentu Y (2020) Polyphyllin I attenuates cognitive impairments and reduces AD-like pathology through CIP2A-PP2A signaling pathway in 3XTg-AD mice. FASEB J 34(12):16414–16431. 10.1096/fj.202001499R 33070372 10.1096/fj.202001499R

[CR152] Zu G, Sun K, Li L, Zu X, Han T, Huang H (2021) Mechanism of quercetin therapeutic targets for Alzheimer disease and type 2 diabetes mellitus. Sci Rep 11(1):22959. 10.1038/s41598-021-02248-5 34824300 10.1038/s41598-021-02248-5 PMC8617296

